# SARS-CoV-2 nucleocapsid protein adheres to replication organelles before viral assembly at the Golgi/ERGIC and lysosome-mediated egress

**DOI:** 10.1126/sciadv.abl4895

**Published:** 2022-01-07

**Authors:** Katharina M. Scherer, Luca Mascheroni, George W. Carnell, Lucia C. S. Wunderlich, Stanislaw Makarchuk, Marius Brockhoff, Ioanna Mela, Ana Fernandez-Villegas, Max Barysevich, Hazel Stewart, Maria Suau Sans, Charlotte L. George, Jacob R. Lamb, Gabriele S. Kaminski-Schierle, Jonathan L. Heeney, Clemens F. Kaminski

**Affiliations:** 1 Department of Chemical Engineering and Biotechnology, University of Cambridge, Cambridge, UK.; 2 Department of Veterinary Medicine, University of Cambridge, Cambridge, UK.; 3 UK Dementia Research Institute, Cambridge, UK.; 4 Division of Virology, Department of Pathology, University of Cambridge, Cambridge, UK.

## Abstract

Despite being the target of extensive research efforts due to the COVID-19 (coronavirus disease 2019) pandemic, relatively little is known about the dynamics of severe acute respiratory syndrome coronavirus 2 (SARS-CoV-2) replication within cells. We investigate and characterize the tightly orchestrated virus assembly by visualizing the spatiotemporal dynamics of the four structural SARS-CoV-2 proteins at high resolution. The nucleoprotein is expressed first and accumulates around folded endoplasmic reticulum (ER) membranes in convoluted layers that contain viral RNA replication foci. We find that, of the three transmembrane proteins, the membrane protein appears at the Golgi apparatus/ER-to-Golgi intermediate compartment before the spike and envelope proteins. Relocation of a lysosome marker toward the assembly compartment and its detection in transport vesicles of viral proteins confirm an important role of lysosomes in SARS-CoV-2 egress. These data provide insights into the spatiotemporal regulation of SARS-CoV-2 assembly and refine the current understanding of SARS-CoV-2 replication.

## Teaser

Super-resolution optical imaging reveals the accumulation of SARS-CoV-2 nucleocapsid protein at viral replication organelles.

## INTRODUCTION

Severe acute respiratory syndrome coronavirus 2 (SARS-CoV-2) is an RNA virus and the causative agent of coronavirus disease 2019 (COVID-19) (*1*). To date, more than 263 million cases of this disease have been diagnosed, resulting in more than 5.4 million deaths (*2*). Great efforts have been made in the development of measures for containing the spread of SARS-CoV-2, including the repurposing of previously produced drugs (*3*), therapies (*4*), and the development of vaccines (*5*).

While new diagnosis, prevention, and treatment options for COVID-19 continue to emerge at a rapid pace, the understanding of the biology of SARS-CoV-2 advances more slowly. Unraveling the mechanisms of transmission and replication of this virus is crucial for the development of rationally designed drugs and vaccines, and to understand the long-term effects of the disease, allowing researchers to develop countermeasures against evolving SARS-CoV-2 variants of concern.

SARS-CoV-2 spreads among humans primarily via respiratory droplets when two individuals are in close proximity (*6*). It is an enveloped virus that enters the cells of the respiratory tract through the interaction of the receptor binding domain on the spike (S) protein and the angiotensin-converting enzyme 2 receptor on the cell surface (*7*). The positive sense, single-stranded RNA genome of SARS-CoV-2 is then released into the host cell cytosol and is directly translated. Two large open reading frames (ORF1a and ORF1ab) are translated into large polyprotein complexes (pp1a and pp1ab), which are cotranslationally and posttranslationally cleaved to generate 16 nonstructural proteins, for which characterization is ongoing (*8*). The remaining ORFs encode the four structural proteins of SARS-CoV-2 (*9*). In coronaviruses in general, the nucleocapsid (N) protein encapsulates the viral RNA (*9*, *10*), the S protein mediates cell entry (*7*), the membrane (M) protein is embedded in the envelope and thought to provide a scaffold for viral assembly (*11*), and the envelope (E) protein forms ion-conductive channels in the lipid viral envelope (*12*). Upon infection by SARS-CoV-2, the virus initiates the biogenesis of replication organelles (ROs) containing interconnected perinuclear double-membrane structures such as double-membrane vesicles (DMVs), which are derived from, and tethered to, the endoplasmic reticulum (ER) (*13*). It is assumed that these structures protect the viral RNA from degradation by cellular ribonucleases and detection by host cellular immune sensors during genome replication (*14*). This hypothesis has been corroborated by the recent finding of Klein *et al.*, who have proven the presence of viral RNA inside DMVs (*15*, *16*). The DMVs have a pore in their double-membrane lining, by which the RNA is thought to access the cytosol (*17*). The assembly of mature SARS-CoV-2 virions occurs within the ER-to-Golgi intermediate compartment (ERGIC) (*8*, *13*). The egress of coronaviruses is assumed to occur via exocytosis (*15*). Recent evidence suggests that newly formed SARS-CoV-2 virions reach the cell periphery using lysosome trafficking (*18*).

The interactions of each SARS-CoV-2 protein with a series of host cell proteins have been partially studied by combining light microscopy and proteomics (*19*, *20*). Gordon *et al.* (*19*) used confocal microscopy to study the distribution of two of the structural proteins of SARS-CoV-2 in infected Caco-2 cells at one time point after infection. The imaging revealed a cytosolic signal for the N protein and strong colocalization of the M protein with the Golgi apparatus. However, the location of the viral proteins during infection is very dynamic and complex because it is highly dependent on their interaction with each other and the host cell, conditions that cannot be simulated by transfection. In the current work, we add to the understanding of SARS-CoV-2 replication by studying the timing and location of the interplay between all four structural proteins of SARS-CoV-2 and the host cell over the time course of the infection cycle.

The replication of SARS-CoV-2 is known to extensively change the localization and reshape the morphology of cell organelles and the cytoskeleton within the host cell. Such morphological alterations have recently been studied in Calu-3 cells using both optical and electron microscopy (*13*). The study by Cortese *et al.* analyzed infected cells at a series of time points after infection to detail the progression of the viral cycle, focusing on the host cell structures in detail. They demonstrated the progressive fragmentation of the Golgi apparatus, the recruitment of peroxisomes to the sites of viral replication, and the reshaping of the vimentin network to accommodate the DMVs. While electron microscopy highlighted cellular structures with high definition, the viral proteins were visualized with lower resolution using confocal microscopy. The power of super-resolution optical microscopy has been demonstrated by the application of three-dimensional (3D) STED (stimulated depleted emission microscopy) to reveal the formation of a vimentin cage around ROs. However, the latter technique cannot be performed in high-throughput fashion and is not easily adapted for multiplexed imaging of several proteins simultaneously.

Here, we use a range of light microscopy techniques, particularly a combination of wide-field, confocal, light sheet, and expansion microscopy, to overcome some of these limitations. To obtain high-quality imaging data, Vero cells were used for infection because their morphology is well suited for fluorescence imaging and single cell analysis. In addition, numerous SARS-CoV-2 studies based on Vero cells exist, allowing us to put our results into context. For this study, a fixation protocol that permits transport of infected Vero cells from class 3 containment laboratories to high-resolution imaging facilities was developed. Establishing immunostaining protocols for the imaging of multiple SARS-CoV-2 and host cell proteins simultaneously provided well-defined and controlled snapshots in up two four colors at subwavelength resolution at different infection stages. We present a detailed investigation of the spatiotemporal organization of the four structural proteins of SARS-CoV-2 within the host cell during an infection cycle. Specifically, we focus on expression kinetics, the dynamic location at different host cell compartments during assembly and egress as well as organelle and cytoskeleton rearrangement that is associated with these processes. The four structural proteins are expressed differentially. On the basis of the expression patterns, classification criteria are defined and three distinct infection stages are identified. Sorting of single cells in these distinct infection stages is used to assess the dynamics of host cell remodeling. We find that reshaping of microtubules, relocation of lysosomes, and fragmentation of the Golgi apparatus largely correlate with the local accumulation of the three viral transmembrane proteins, S, E, and M proteins. By combining expansion microscopy and light sheet microscopy, we have produced volumetric maps of protein distributions in whole infected cells. In particular, we see that the N protein associates with convoluted and fused membrane compartments. The N protein accumulates in the outer layers of those compartments that fold around ER membranes and contain at least one double-stranded RNA (dsRNA) focus, suggesting that they are viral ROs (vROs).

## RESULTS

### The cellular distribution of SARS-CoV-2 structural proteins is tightly regulated in space and in time

We first optimized cellular fixation and staining protocols, using transfection to express the four structural proteins of SARS-CoV-2 individually in Vero cells. We also optimized fixation (formaldehyde and glyoxal) and permeabilization (Triton X-100 and saponin) reagents. Each cellular structure has its own ideal immunostaining conditions (fig. S1); the ER was best fixed in a glyoxal buffer, preserving the fine structure of the tubular regions. In contrast, the Golgi apparatus was only stained when fixed with formaldehyde independently of the detergent. As a final example, lysosomal staining was only achieved after permeabilization with saponin. The optimal staining conditions for the cellular structures being investigated determined the choice of experimental conditions for each sample. A summary of the optimized fixation and permeabilization conditions for each of the structures investigated in this work is presented in table S1.

The immunostaining of the transfected cells with the selected antibodies was successful in all fixation and permeabilization conditions tested (fig. S2). The pattern of the S protein staining was different in the two fixation conditions (formaldehyde and glyoxal). However, we did not note any differences when fixing and staining infected cells in these two conditions. It has been previously observed that the intracellular localization of viral proteins can differ considerably when comparing an individually expressed viral protein and the same protein within an infected cell (*19*). These observations confirmed that investigations should be carried out in virus-infected cells.

In this study, we fixed infected cells at multiple time points after infection (5, 7.5, 10, 12, and 24 hours). The spatial distribution of the viral proteins changed over time and varied between individual cells at the same time after infection, particularly at later time points. A high degree of cell-to-cell variability in infection is expected and has been observed for a range of mammalian viruses. Known sources for this variability can be the difference in the number of virions that cells encounter, genetic variation of virus particles, and the state of the host cell during infection. However, we identified similarities in the expression and distribution of the viral proteins in individual cells within the heterogeneous population and across time points. These patterns correspond to distinct events in the replication cycle. We used these patterns to classify the cells into different categories. This analysis on single cells rather than the population average proves useful as it allows us to gain a clearer picture of how the virus cycle is staged in time and to connect certain steps in the viral replication cycle with morphological changes in the host cell.

**Fig. 1.**
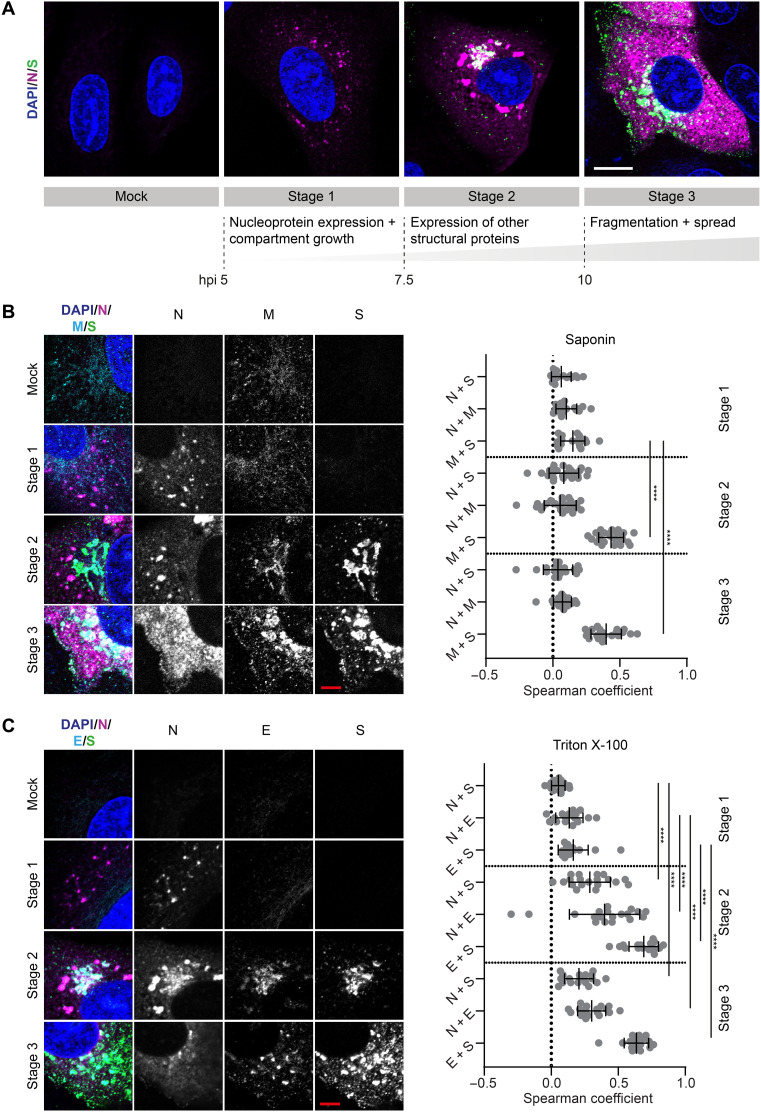
The cellular distribution of structural proteins of SARS-CoV-2 is tightly regulated in space and in time. (**A**) Three categories describing different cell states were identified on the basis of the distribution pattern of the N and S proteins during the late phase of the SARS-CoV-2 replication cycle. These categories were termed stages 1, 2, and 3, respectively. For each stage, a representative confocal microscopy image is shown. Blue: DAPI (4′,6-diamidino-2-phenylindole)–stained nuclei; magenta: N protein; green: S protein. Scale bar, 10 μm. (**B**) Colocalization between SARS-CoV-2 N, M, and S proteins. Cells were fixed with glyoxal and permeabilized with saponin. Left: Representative confocal images of infected Vero cells in infection stages 2 and 3. Blue: DAPI-stained nuclei; magenta: N protein; cyan: M protein; green: S protein. Scale bar, 5 μm. Right: Colocalization between viral proteins N, S, and M at different infection stages determined using the Spearman coefficient method (stage 1: *n* = 20; stage 2: *n* = 25; stage 3: *n* = 20). Manders coefficients are shown in fig. S4A. (**C**) Left: Representative confocal images of SARS-CoV-2–infected Vero cells in infection stages 2 and 3. Cells were fixed with formaldehyde and permeabilized with Triton X-100. Blue: DAPI-stained nuclei; magenta: N protein; green: S protein; cyan: E protein. Scale bar, 5 μm. Right: Colocalization between viral proteins N, S, and E at different infection stages determined using the Spearman coefficient method (stage 1: *n* = 16; stage 2: *n* = 19; stage 3: *n* = 18). Manders coefficients are shown in fig. S4B. Significance was tested for all datasets with an unpaired Mann-Whitney test. See figs. S5 and S6 for more exemplary images.

We found that three different categories or stages were sufficient to classify the status of any cell in the population (Fig. 1A). At an early infection stage [5 hours post-infection (hpi)], cells were seen to express the N protein only. At this stage (referred to as stage 1), the N protein is not homogeneously distributed inside the host cell cytosol but forms small puncta. From 7.5 hpi onward, the other structural proteins of SARS-CoV-2—S, M, and E proteins—are expressed and localized in a compact juxtanuclear membrane compartment (stage 2). Cells that were characterized by fragmentation and spreading of the compartments containing the S, M, and E proteins were classified as stage 3; in these cells, the N protein is highly dispersed in the cytoplasm. At stage 3 (abundantly present from 10 hpi onward), we observed small dots of all four structural viral proteins at the plasma membrane, indicating the formation and trafficking of mature SARS-CoV-2 virions. These relative timings within the replication cycle were not previously known in this detail. The findings were enabled by the classification strategy described here, which considers the staging of cells individually rather than population averages at different times after infection.

The selection of antibodies against the structural proteins allowed us to image three of the four structural proteins at once. The M and E protein could not be visualized simultaneously because the respective antibodies belonged to the same host species. Instead, we immunostained two separate sets of samples, costaining either for N, M, and S proteins (Fig. 1B) or for N, E, and S proteins (Fig. 1C). We expected the M and E proteins to show a similar pattern of accumulation in the same host cell membrane compartment as the S protein, as these are all transmembrane proteins. Representative images and quantitative colocalization analysis confirmed that M (Fig. 1B) and E proteins (Fig. 1C) co-occurred foremost with the S protein and not the N protein. Consequently, M and E proteins follow the same accumulation and fragmentation pattern as the S protein.

By comparing the colocalization values between N and transmembrane proteins (M, S, and E proteins) within the two separate sample sets for stages 2 and 3, we noticed that, whereas the Spearman coefficients (see the “Colocalization analysis” section) were close to zero in the first sample set (Fig. 1B), they were increased in the second sample set (Fig. 1C). This is not due to a different localization pattern of the proteins in the separate sample sets but due to a different capability to visualize the viral proteins depending on the permeabilization reagents used for immunostaining. The use of strong (Triton X-100) instead of mild (saponin) detergents was required to visualize the N protein at the juxtanuclear membrane compartment, in addition to the bright N protein puncta, where the transmembrane proteins also localize. The stronger detergent also leads to an increased staining of cytoplasmic, dispersed N protein (fig. S3). The colocalization of N protein with the three transmembrane proteins at this compartment is in line with the current model for SARS-CoV-2 assembly, where viral nucleocapsids are trafficked to membrane compartments enriched with M, S, and E proteins for assembly.

### The kinetic profile of SARS-CoV-2 replication

**Fig. 2.**
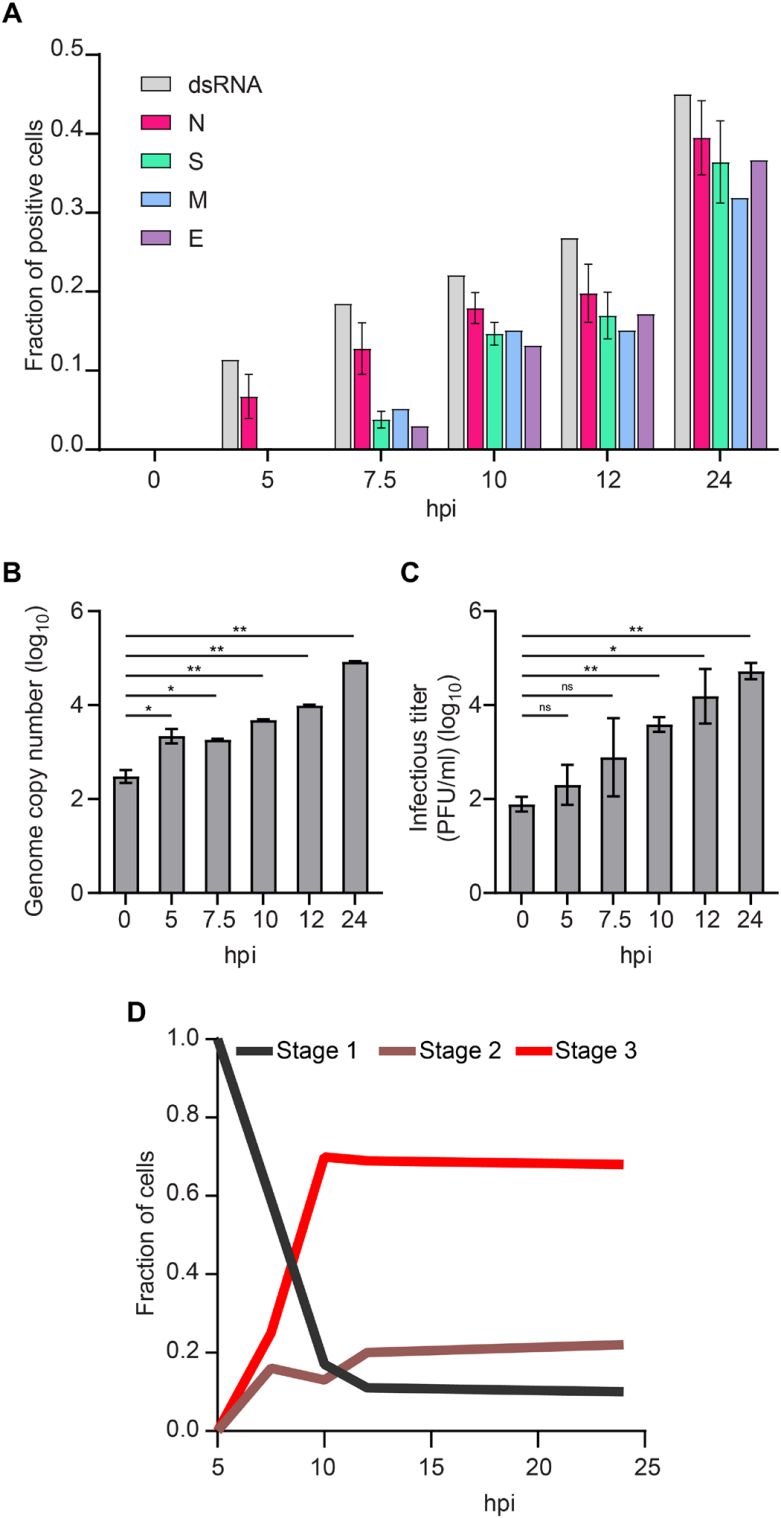
Stepwise expression of the four structural SARS-CoV-2 proteins and dsRNA correlates with staged release of viral transcripts (5 hpi) and infectious virus (7.5 to 10 hpi), respectively. (**A**) The fraction of cells positive for each of the structural proteins of SARS-CoV-2 or viral dsRNA was determined by immunostaining of infected Vero cells. For each time point, 30 to 35 wide-field microscope images, corresponding to 1000 to 1500 cells per sample, were analyzed. For the control time point of 0 hpi, around 250 cells were analyzed per sample. For the count of N- and S-positive cells, three samples per time point were analyzed. (**B**) The copy number of the viral transcripts in the cell supernatant was measured by RT-qPCR. Release of viral RNA was observed from 5 hpi onward when first cells started expressing N protein. (**C**) The infectious titer of the cell supernatant was determined by the plaque assay. Newly formed infective SARS-CoV-2 virions were released from cells from 10 hpi onward. For both assays, two replicates were carried out. Significance was tested with an unpaired *t* test. (**D**) Stage-dependent replication kinetics. ns, not significant.

To assess SARS-CoV-2 replication kinetics, we determined the fraction of cells expressing each of the four structural proteins and the fraction of cells in which dsRNA was present (indicating initiation of viral RNA transcription) at each time point (Fig. 2A). At 5 hpi, 5 to 10% of cells were positively stained for dsRNA and N protein, but none of the other structural proteins. Consistently, we detected released viral transcripts in the cell supernatant by quantitative real-time transcription polymerase chain reaction (RT-qPCR) from 5 hpi onward (Fig. 2B). These data confirm the observations of Cortese *et al.* in Calu-3 cells, where PCR, an infectivity assay, and transmission electron microscopy (TEM) were used. In the latter report, the release of viral RNA and infectious virus was observed in parallel with the appearance of DMVs at 6 hpi under similar experimental conditions (*13*). This suggests that the timing of events during viral replication is comparable between Calu-3 and Vero cells. At 7.5 hpi, we observed that the fraction of cells positive for N protein increased by up to ~15%, with about a third of the cells also expressing the other three structural proteins. From 10 hpi on, infected cells were expressing all four structural proteins at similar levels. This is again consistent with a significantly increased infectious titer at 10 hpi (Fig. 2C), confirming the completion of the replication cycle and the production of new viruses. For most cells, it appeared that M and E proteins were expressed simultaneously with the S protein. However, in a few cells, only fluorescence signal from the M, but not the S, protein was detected (~5% of infected cells; see representative cell in fig. S7). In contrast, E and S proteins always occurred together. This indicates that the M protein is expressed before S and E proteins. At 24 hpi, we observed a doubling in the number of infected cells compared to 12 hpi. We then tracked the average expression level over time by measuring the average fluorescence intensity per cell (fig. S8). For all four viral proteins, the trend was similar: The average expression levels per cell increased until 12 hpi when they saturated. While the average values at 12 and at 24 hpi are comparable, we note that the distributions of values are more homogeneous at 24 hpi than they are at 12 hpi.

Last, we classified the cells, according to them being in three different stages, to quantify the kinetic profile of the infection process (Fig. 2D). Between 5 and 10 hpi, we saw a strong shift from stage 1 to stages 2 and 3. At 7.5 hpi, 50% of cells express all four structural proteins, with an equal number of cells observed in stages 2 and 3 (compact versus fragmented juxtanuclear membrane compartment). From 10 hpi on, we observe a rapid increase in cells with fragmented compartments (stage 3) that are dominating the population of infected cells (~75%), whereas the fractions of cells in stages 1 and 2 remain low. This indicates that the transition from stage 2 to stage 3 (compact to fragmented juxtanuclear membrane compartment) mainly occurs between 7.5 and 10 hpi. This transition also coincides with a significantly increased production of mature virions at 10 hpi (Fig. 2C).

### The N protein accumulates around folded ER membranes in convoluted layers that connect to viral RNA replication foci

**Fig. 3.**
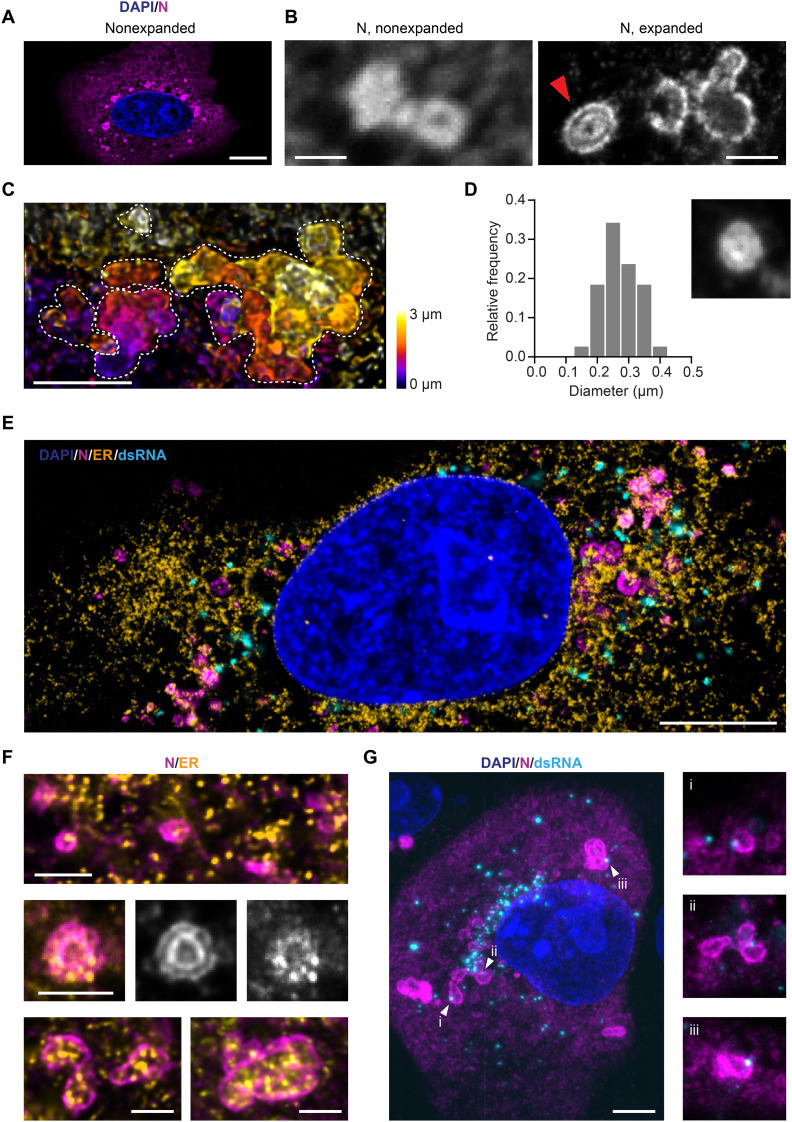
The SARS-CoV-2 N protein is organized in layered structures that are strongly interwoven with the topology of the ER and contain RNA replication foci. (**A**) Confocal microscopy cannot resolve the substructure of N protein puncta in the cytosol of infected Vero cells (blue: nuclei; magenta: N protein). Scale bar, 10 μm. (**B**) The combination with expansion microscopy reveals double layers of N protein within the larger compartments (red arrow). Scale bars, 1 μm. Linear expansion factor of 4.2. (**C**) Combining expansion with light sheet microscopy reveals the convoluted nature of the N protein structures in 3D. Dotted lines outline the boundaries of each convoluted compartment in the maximum intensity projection. Scale bar, 2 μm. Expansion factor of 4.2. (**D**) The inner circular layer of the N protein double-layer compartments has an average diameter of 275 nm. Thirty-eight compartments from 10 cells at 12 hpi were analyzed. The micrograph size is 1.25 μm per side. (**E**) Representative confocal image of an expanded, infected Vero cell stained for the nucleus (blue), ER (calnexin; orange), N protein (magenta), and dsRNA (cyan). Scale bar, 5 μm. Expansion factor of 4.2. (**F**) The N protein (magenta) forms layers around ER membranes (yellow). Scale bars, 1 μm. Expansion factor of 4.2. (**G**) A combination of expansion and light sheet microscopy reveals that dsRNA foci (cyan) sit in the layers of the N compartments (magenta). Scale bar, 5 μm. Expansion factor of 4.2. The size of the smaller micrographs is 5 μm per side.

As shown in Fig. 1, the intracellular location of the N protein is distinct from that of the other three structural proteins: Initially, the N protein accumulates exclusively in small puncta; as the infection progresses, cytosolic signal gradually increases alongside the puncta (Fig. 3A). In parallel, the number, as well as the size, of N protein puncta grow significantly (fig. S9). At closer inspection of the larger puncta in images of infected cells fixed at 10 hpi, the round structures were found to be shaped like vesicles with an outer layer containing N protein and a hollow center (Fig. 3B, left image). We propose that these N protein layers are formed at the vROs.

To find support for this hypothesis, we applied expansion microscopy (*21*) to investigate these structures in better detail. This super-resolution technique provides an at least fourfold increase in resolution via a 64× volumetric expansion of the sample. In these higher resolved images of the N puncta, we detected that several of the N compartments consisted of double layers of the protein (Fig. 3B, right image, and movie S1). In addition, we observed that single small compartments were often fused to larger convoluted 3D structures (Fig. 3C and movies S2 and S3).

The inner N protein compartments measured ~275 nm on average in diameter (Fig. 3D). vROs contain single DMVs and DMV packets (vesicle packets) (*15*). DMVs formed by SARS-CoV-2 contained in the ROs are about 300 nm in diameter (*13*, *15*), which is in agreement with the structures presented. It is accepted that the DMVs formed by coronaviruses are used by the virus as a protective environment for replication of its RNA genome (*14*, *15*), and the presence of SARS-CoV-2 RNA in such structures was recently verified by electron microscopy (*15*).

It has been established that ROs are derived from ER membranes and serve as an anchor for the viral replication and transcription complex (RTC) (*22*). dsRNA is considered a viral replication intermediate, indicating the proximity of RTCs. By costaining the N protein, ER, and dsRNA, and then acquiring confocal images of nonexpanded cells (fig. S10, A and B) as well as expanded cells (Fig. 3E), we found that, at all stages of infection, the N protein–containing compartments were always associated with the ER. Moreover, the ER membranes seemed clustered at the spots where those compartments are present. In the expanded samples, we observed that the N protein formed layers around the highly convoluted ER membranes (Fig. 3F). This was observed for single small (<1 μm), larger fused (>1 μm), as well as double-layered N protein compartments.

Analogously to the N protein–enriched compartments, the dsRNA foci were also always associated with the ER (Fig. 3E). Only some of the N protein–enriched compartments seemed to colocalize with dsRNA foci, whereas many replication foci were not co-occurring with the N protein compartments. A quantitative analysis of confocal images of nonexpanded cells showed that the fraction of closely associated compartments and foci decreased for cells in later infection stages (fig. S11), when the number of dsRNA foci increased (fig. S10B).

However, single-image cell sections might be misleading, as they omit information on either side of the focal plane. To analyze the connection between dsRNA foci and N protein–layered compartments, we acquired volume sections of 13 expanded cells using light sheet microscopy (movie S4). In most samples, most dsRNA foci are located in a region immediately adjacent to the nucleus. We further noted that most N protein compartments were connected to at least one RNA replication focus, which was usually situated in the outer layer of the compartment (Fig. 3G).

### The S protein accumulates in Golgi/ERGIC compartments and transport vesicles containing a lysosome marker

Next, we aimed to determine with which organelles the SARS-CoV-2 transmembrane proteins directly interact during assembly and egress. Because of the selection and limitation of the used antibodies, we could only visualize S protein simultaneously with the host cell structures. However, the three transmembrane proteins S, M, and E show a high degree of colocalization, making it likely that they behave in a similar fashion.

**Fig. 4.**
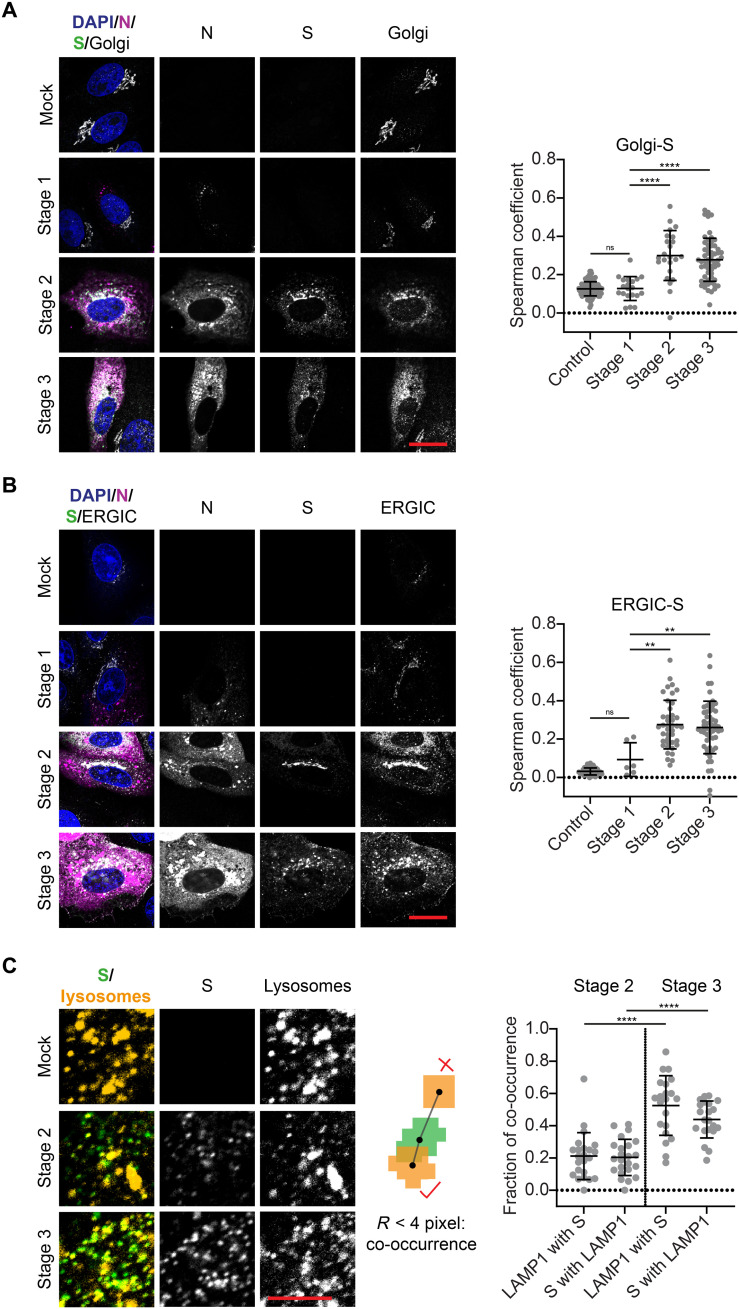
The S protein accumulates at the Golgi apparatus as well as the ERGIC and colocalizes with lysosomes during late infection stages. (**A**) Representative confocal images of SARS-CoV-2–infected Vero cells in infection stages 2 and 3 stained for nuclei (blue), N protein (magenta), S protein (green), and Golgi apparatus (GM130; cyan). Scale bar, 20 μm. See fig. S12 for more exemplary images. Colocalization analysis shows partial spatial correlation between S protein and the Golgi apparatus when S protein expression is detected (from stage 2 onward). Control: *n* = 111; stage 1: *n* = 20; stage 2: *n* = 21; stage 3: *n* = 59. See fig. S13A for Manders coefficients. (**B**) Representative confocal images of SARS-CoV-2–infected Vero cells in infection stages 2 and 3 stained for nuclei (blue), N protein (magenta), S protein (green), and ERGIC (LMAN-1; cyan). Scale bar, 20 μm. See fig. S14 for more exemplary images. Partial spatial correlation between S protein and the ERGIC is detected from stage 2 onward. Control: *n* = 44; stage 1: *n* = 6; stage 2: *n* = 41; stage 3: *n* = 58. See fig. S13B for Manders coefficients. (**C**) Representative confocal images of SARS-CoV-2–infected Vero cells stained for S protein (green) and lysosomes (LAMP1; yellow). Scale bar, 5 μm. Spot-to-spot distance analysis reveals increased fraction of co-occurring S protein and lysosome spots at stage 3 compared to stage 2. Significance was tested with an unpaired *t* test with Welch’s correction for unequal SDs.

The S protein was seen to be at least partially located in the Golgi apparatus and ERGIC from a co-occurrence with the respective organelle markers 130 kDa cis-Golgi matrix protein (GM130) and lectin mannose protein 1 (LMAN-1) during stages 2 and 3 (Fig. 4, A and B). This finding corresponds with observations made previously also for SARS-CoV-1 (*23*). We quantified this colocalization by determining the Spearman coefficients, which exhibited average values of ~0 in control cells and in cells at stage 1 but increased significantly at stages 2 and 3 in all cases.

Concurrent to the enrichment of S protein at Golgi and ERGIC membranes at stage 2, small spots of S protein appeared in the cytoplasm (Figs. 1A and 4A). This indicates trafficking of the S protein, and supposedly also M and E proteins, away from the Golgi and ERGIC membranes. These diffraction-limited spots could either be transport vesicles containing viral proteins in their lipid membranes or newly formed virions. It has recently been reported that lysosomes are used by the virus to exit the cell and that mature virions exploit this for transport to the cell surface (*18*). When we stained the cells for the lysosome marker lysosome-associated membrane glycoprotein 1 (LAMP1), we found that the spots containing S protein were often also positive for LAMP1 (Fig. 4C). We quantified co-occurrence of S protein and LAMP1 for stages 2 and 3 using a spot-to-spot distance analysis. When the centers of spots in both channels were within a distance of 280 nm, they were considered as co-occurring. The fraction of co-occurring spots increased from ~20 to ~50% for both S protein and lysosomes at stage 3. These findings confirm that lysosomes can be used for the shuttling of virions, further supporting the role of lysosomes in SARS-CoV-2 egress.

### The infection alters the morphology and location of host cell organelles and cytoskeleton

**Fig. 5.**
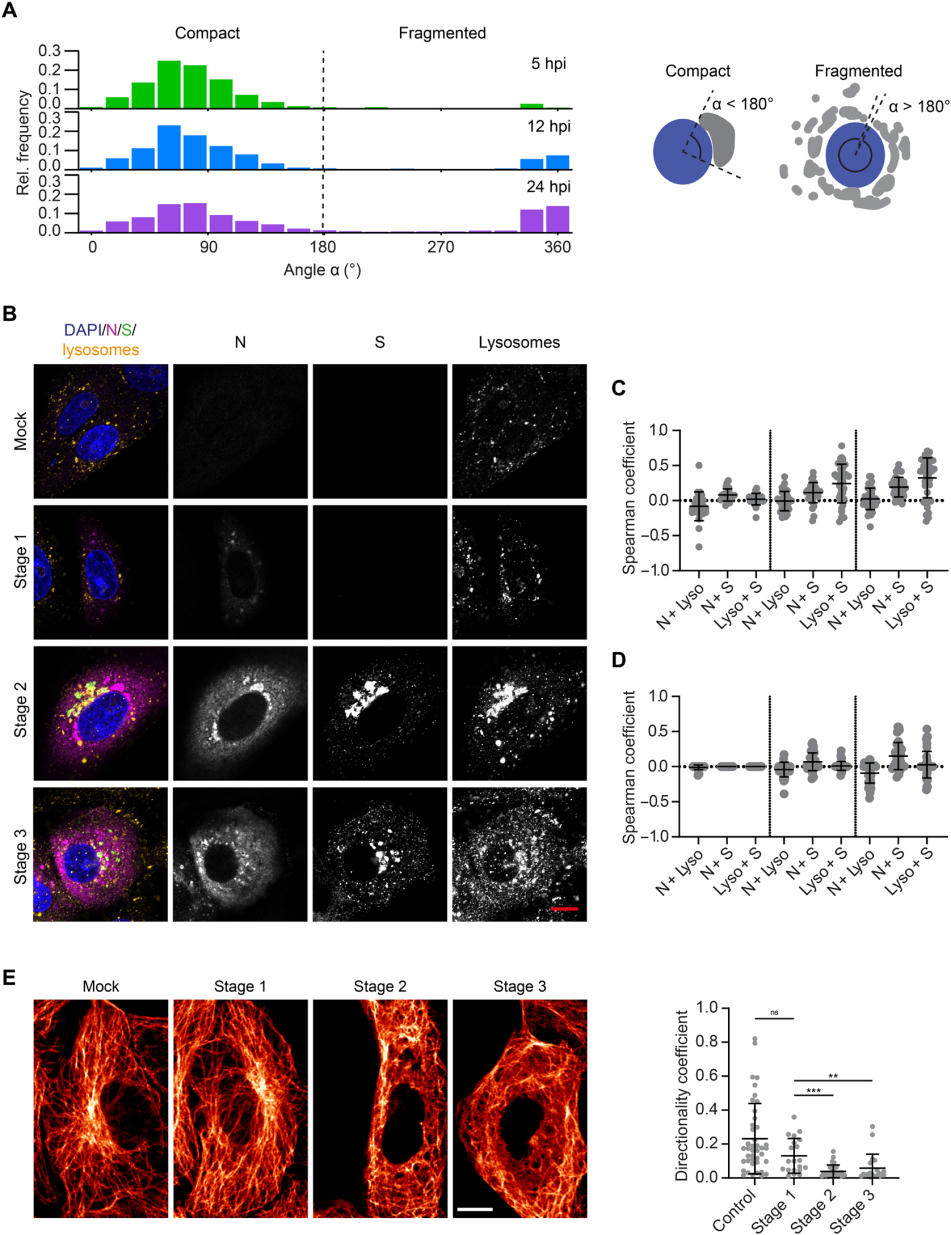
SARS-CoV-2 infection alters the morphology and location of organelles and the cytoskeleton in Vero cells. (**A**) The angular distribution of Golgi compartments around the nucleus was used to distinguish between cells with compact (angle < 180°) and fragmented Golgi apparatus (angle > 180°). The fraction of cells with fragmented Golgi apparatus increased over the time course of infection. The fragmentation of the Golgi apparatus upon SARS-CoV-2 infection was used to sort cells into infection stage 2 or 3 (Fig. 2D). (**B**) Left: Representative confocal images of lysosomes in infected Vero cells at different infection stages. Blue: DAPI-stained nuclei; magenta: N protein; green: S protein; yellow: lysosomes (LAMP1 staining). Scale bar, 10 μm. See fig. S15 for more exemplary images. Right: Colocalization of the lysosome marker LAMP1 with the N and S proteins. (**C**) Otsu thresholding allows correlation between all LAMP1 and SARS-CoV-2 protein signal. (**D**) Manual thresholding, however, filters out the weaker LAMP1 signal such that only bright, small lysosomal compartments are taken into account (stage 1: *n* = 25; stage 2: *n* = 34; stage 3: *n* = 39). (**E**) Left: Representative confocal images of microtubules in control (mock infected) and infected Vero cells (stages 1, 2, and 3). Scale bar, 10 μm. See fig. S16 for more exemplary images. Right: The directionality coefficient was calculated for subareas of the microtubule network. Each data point corresponds to one subregion inside a cell. Changes in the network because of SARS-CoV-2 infection lead to significant reduction in directionality at stage 1, which is even more pronounced at stages 2 and 3 when all four structural proteins are expressed. Significance was tested with a Mann-Whitney test.

We further analyzed the morphological changes of the host cell organelles involved in SARS-CoV-2 assembly and egress as well as the cytoskeleton at different stages of infection. The most notable morphological change that we noted was a fragmentation of the Golgi compartment. To quantify this fragmentation, we measured the angle spanned by the Golgi apparatus around the nucleus, as depicted in Fig. 5A. In cells with fragmented compartments, the angles were typically larger than 180° and often close to 360°. Thus, we distinguished between cells with a compact (<180°) or fragmented (>180°) Golgi compartment. The histograms represent the distributions of angles measured in the cell population at different times after infection. At 5 hpi, we observed almost no fragmentation. At 12 and 24 hpi, however, the fraction of cells with fragmented Golgi compartments was increased. This corresponds to cells in late infection stages (stage 3), when new mature viruses were being produced and released.

We also noticed that the lysosomes undergo a spatial redistribution during SARS-CoV-2 infection (Fig. 5B). At stage 1, the lysosomes were larger on average than in control cells (control: mean area = 0.94 μm^2^, *n* = 56; stage 1: mean area = 1.22 μm^2^, *n* = 21) but were still homogeneously distributed within the cytoplasm. When cells started to express S, M, and E proteins (stage 2), the lysosome marker LAMP1 was recruited to the Golgi/ERGIC compartments containing the three viral transmembrane proteins, likely through fusion of the lysosomes with the Golgi and ERGIC membranes. The fragmentation of the Golgi apparatus (stage 3) corresponded to a spread of membrane fragments enriched with viral proteins and LAMP1 in the cytoplasm. The correlation of the lysosome signal with the viral proteins N and S (measured via the Spearman coefficient after Otsu thresholding) was moderate at stages 2 and 3 (Fig. 5B). There are two distinct sources from which the lysosome signal originates: small and bright compartments resembling the typical lysosome shape (Fig. 5C) and LAMP1 accumulated at the Golgi and ERGIC membranes (Fig. 5D), but with markedly lower intensity. We used manual thresholding to filter out the weaker signal and only investigate the correlation between the small, bright lysosomal compartments with the viral proteins. We detected no correlation between S protein and the lysosomes in that case. Furthermore, there was a negative correlation between N and the lysosomes at stage 3. At this stage, the N protein was widely distributed in the cytoplasm but excluded from the location of the lysosomes. This indicates that the correlation between the viral proteins and LAMP1 only occurs at the compartments after a mixing of membranes.

SARS-CoV-2 infection leads to a remodeling of the microtubule network. Through a directionality analysis, we found that, from stage 2 onward, the network loses its orientation (Fig. 5E). In noninfected cells, microtubules spread from the microtubule-organizing center close to the Golgi apparatus to the extremities of the cell. In late infection stages, the microtubule filaments were absent from the juxtanuclear area and they were entangled when compared to control cells. For cells in stages 2 and 3, we also detected a loss of cell stiffness, which we measured using atomic force microscopy (AFM; fig. S17). This could be driven by a remodeling of the actin network, which is regarded as the overriding, although not the sole, determinant of cell stiffness (*24*).

## DISCUSSION

We applied advanced fluorescence microscopy to investigate the expression kinetics and spatial arrangement of the four structural SARS-CoV-2 proteins and studied their colocalization with host cell compartments in detail. We observed that the expression of the structural proteins of the virus is tightly staged, with notable differences between N and the three transmembrane proteins. The N protein accumulates mainly in small foci that grow in size and number during the course of infection. Sample expansion in combination with light sheet microscopy revealed that single N protein compartments comprise layered structures of N protein. The compartments resemble complex and convoluted 3D structures as might result from the fusion and engulfment of smaller vesicular subunits. We believe them to be part of the ROs formed by SARS-CoV-2. There are several indicators to support this notion. First, the shapes of the N protein compartments resemble those of ROs investigated by electron microscopy, where interconnected DMVs and vesicle packets were observed (*15*). Second, the smallest structural units we could identify within these convoluted structures were vesicles whose average size was ~275 nm, which matches the size reported for DMVs in Vero cells (*15*). Third, it is known that coronaviruses remodel the host cell ER membranes to integrate the vROs (*25*, *26*). We found that the N protein–containing compartments are tethered to ER membranes. Last, we reasoned that if the N protein was associated to the vROs, the N compartments would be associated with the viral RTCs. We detected the RTCs by staining of dsRNA, an intermediate of viral RNA replication. It needs to be mentioned that the amount of genomic viral RNA that is present in infected cells is underestimated by using a dsRNA antibody that indicates only replication factories with high dsRNA content (*27*). Through volumetric imaging, we confirmed that at least one dsRNA focus is usually associated with the outer layer of an N protein compartment, which might consist of several fused sections. We also noted that, similarly to the N protein compartments, the dsRNA foci are always connected to the ER network. For viral genomic RNA compared to dsRNA, a stronger colocalization with the N protein would be expected. Using in situ hybridization assays for visualizing viral genomic RNA, Stertz *et al.* (*16*) for SARS-CoV and Lee *et al.* (*27*) for SARS-CoV-2 could indeed demonstrate a high co-occurrence of N protein and viral RNA in infected cells.

Because one of the functions of the N protein is the encapsulation of the viral RNA, its presence at/around the DMVs and colocalization with proteins forming the RTCs would not be unexpected in accordance with previous reports for SARS-CoV (*16*). However, to our knowledge, association of N protein to ROs has not been reported before. But it seems to make sense from the virus perspective since such an arrangement would spatially connect replication and nucleocapsid formation.

If we assume that the N protein–containing structures are indeed part of the vROs, the question remains where exactly the N protein is located within those compartments and how this association is formed. It is possible that the protein accumulates in the intermembrane space of the DMV envelope. Another possibility is the accumulation of the N protein at ER membranes while or after they are reshaped into ROs. Nonstructural proteins of coronaviruses are known to reshape host cell membranes to induce the formation of DMVs (*14*). Either a specific interaction with one or more SARS-CoV-2 nonstructural proteins or a curvature-driven binding mechanism could drive an accumulation of N protein. In both cases, accumulation might be affected by a propensity of N protein to phase-separate with RNA (*28*–*33*). It has been proposed that N protein plays a dual role: The unmodified protein forms a structured oligomer suitable for nucleocapsid assembly, while the phosphorylated protein forms a liquid-like compartment for viral genome processing (*34*). For both processes, association of N protein to the ROs would thus be beneficial.

A study based on cryo–electron tomography (ET) showed that strands of naked viral RNA are located within the DMVs (*25*), which are thought to leave the DMVs through a pore in the membrane lining (*17*). However, it is currently not known where and when the newly synthesized viral RNA is encapsidated by the N protein. In the light of the data presented here, we speculate that association of the viral RNA and the N protein to form viral ribonucleocapsid protein complexes (vRNPs) occurs at the membrane of the vROs. This process might occur either before or in concurrence with the release of the newly synthesized RNA into the cytosol. In this sense, we interpret the increasing cytosolic signal of the N protein in late infection stages as an accumulation of vRNPs in the cytosol before and during virus assembly.

Using multicolor imaging and colocalization analysis, we show that the SARS-CoV-2 S, M, and E proteins all localize at the Golgi and ERGIC compartments (identified by protein markers) in agreement with previous reports (*8*, *13*). These compartments are also thought to be places of viral assembly (*35*). Using in situ cryo-ET, formation of SARS-CoV-2 virus particles has been observed in regions with a high vesicle density and close to ER- and Golgi-like membrane arrangements (*15*). In addition, vesicles containing assembled virus particles as well as budding events at and into those vesicles were furthermore captured by cryo–FIB/SEM (focused ion beam/scanning electron microscopy) and high-resolution cryo-ET (*36*). This fits well with the picture of assembly that results from our light microscopy data. Accumulation of the N protein at the perinuclear compartment upon expression of S, M, and E strongly indicates these membranes as points of assembly.

Our study showed furthermore that M protein is recruited to this area slightly earlier than S and E proteins, suggesting a predominant role of M protein for controlling the spatial organization of the transmembrane proteins and initiating the assembly of SARS-CoV-2. For other coronaviruses, it has indeed been shown that interactions between M proteins form a lattice into which the other two transmembrane proteins of the virus are incorporated (*37*–*39*). Moreover, we detected that the N protein partially accumulates at the Golgi region, but only after the expression of the other three structural proteins has taken place. It is known that the assembly of coronaviruses is dependent on the M and E proteins, and for SARS-CoV also on the N protein (*39*). In particular, the carboxyl tail of the SARS-CoV-1 M protein interacts specifically with the N protein (*40*). Our results suggest that, also for SARS-CoV-2, the M protein is responsible for the recruitment of the N protein to the Golgi/ERGIC membranes.

At late stages of infection, we detected an enrichment of the lysosomal protein LAMP1 at the membrane compartments together with the structural transmembrane proteins of SARS-CoV-2. This suggests a mixing of membranes or a shift/modification in the endolysosomal transport pathways. Immediately after, diffraction-limited spots of S protein can be seen in the cytoplasm. These spots often co-occur with the lysosome marker. Our results corroborate the recent finding that lysosomes are used by coronaviruses for their cell egress. Ghosh *et al.* (*18*) have observed vesicles containing single SARS-CoV-2 virions with hallmarks of lysosomes using TEM. It is not clear whether the fluorescent spots we observe consist only of vesicles enriched with viral transmembrane proteins or mature virions. Nevertheless, the co-occurrence of viral proteins and lysosome markers indicates an immediate onset of the egress pathway after the expression of the M, S, and E proteins. However, we detected an increase from ~20 to ~50% co-occurrence at transition from a compact (stage 2) to a fragmented Golgi apparatus (stage 3), indicating a surge in viral egress. It is not clear what causes the fragmentation of the Golgi apparatus. It might be caused by an indirect toxic effect due to the accumulation of viral proteins by merging of lysosomes with Golgi membranes and/or the manipulation of the microtubule network, which plays an important role in shaping Golgi structure and function (*41*). We found support for the latter by measuring a remarkable rearrangement of the microtubule network after expression of the three SARS-CoV-2 transmembrane proteins, but before Golgi fragmentation occurs. However, further work is needed to elucidate which factors contribute to the defect in the organization of the Golgi compartments. We envisage that the methods presented in this study could furthermore be used for studying the role of the nonstructural proteins of SARS-CoV-2, the kinetics of the viral genome replication, as well as the relationship between the viral RNA, the N protein, and the vROs.

## MATERIALS AND METHODS

### Biosafety

SARS-CoV-2 infection of cells was conducted at containment level 3. Inactivation of SARS-CoV-2 through fixation was validated using previously published protocols (*42*). The results of this experiment were reviewed and approved by the biosafety committee of the Department of Chemical Engineering of the University of Cambridge.

### Chemicals

Methanol-free formaldehyde was purchased from Thermo Fisher Scientific; the ampoules were used immediately after opening, and any leftover formaldehyde was discarded. Glyoxal (40% in water) was purchased from Sigma-Aldrich; the glyoxal solution was heated and mixed before use to solubilize precipitated glyoxal. Saponin, Triton X-100, and ammonium chloride were purchased from Sigma-Aldrich. All chemicals used for sample expansion (glutaraldehyde, 50% in water, sodium acrylate, *N*,*N*′-methylenbisacrylamide, and acrylamide) were purchased from Sigma-Aldrich and used as received. Lyophilized proteinase K was purchased from Thermo Fisher Scientific. Atto 590–conjugated phalloidin was purchased from Sigma-Aldrich and solubilized in methanol according to the manufacturer’s instructions.

### Antibodies

All the antibodies used in this study are reported in Tables 1 and 2.

**Table 1.** Primary antibodies. IgG, immunoglobulin G.

**Antibody**	**Supplier**	**Target**	**Host species**	**Dilution**
ab273073	Abcam	S protein	Human	1:400
NB100-56569	Novus Biologicals	M protein	Rabbit	1:200
NBP2-41061	Novus Biologicals	E protein	Rabbit	1:200
MA1-7403	Thermo Fisher Scientific	N protein	Mouse IgG2b	1:20
Ab01299-2.0	Absolute Antibody	dsRNA	Mouse IgG2a	1:200
ab22649	Abcam	GM130	Rabbit	1:50
ab125006	Abcam	LMAN-1	Rabbit	1:50
ab24170	Abcam	LAMP1	Rabbit	1:100
ab22595	Abcam	Calnexin	Rabbit	1:200
ab131205	Abcam	β-Tubulin	Mouse IgG1	1:200

**Table 2.** Secondary antibodies.

**Antibody**	**Supplier**	**Target**	**Conjugate**	**Host species**	**Dilution**
A-11013	Thermo Fisher Scientific	Human IgG	Alexa Fluor 488	Goat	1:200
A-11011	Thermo Fisher Scientific	Rabbit IgG	Alexa Fluor 568	Goat	1:200
A-11031	Thermo Fisher Scientific	Mouse IgG	Alexa Fluor 568	Goat	1:100
A-21144	Thermo Fisher Scientific	Mouse IgG2b	Alexa Fluor 568	Goat	1:200
A-21244	Thermo Fisher Scientific	Rabbit IgG	Alexa Fluor 647	Goat	1:100
40839	Merck	Rabbit IgG	Atto 647N	Goat	1:200
50185	Merck	Mouse IgG	Atto 647N	Goat	1:200
610-156-040	Rockland	Mouse IgG1	Atto 647N	Goat	1:100
610-156-041	Rockland	Mouse IgG2a	Atto 647N	Goat	1:100

### Cells and viruses

Vero cells (American Type Culture Collection, CCL-81) were cultured under standard conditions (37°C and 5% CO_2_) in Dulbecco’s minimum essential medium (DMEM) (Sigma-Aldrich) supplemented with 10% heat-inactivated fetal bovine serum (Gibco), antibiotics/antimycotics [penicillin (100 U/ml), streptomycin (100 μg/ml), and Gibco amphotericin B (0.025 μg/ml) Gibco], and 2 mM l-glutamine (GlutaMAX, Gibco). Cells were cultured in T-75 polystyrene flasks; splitting took place when cultures reached ~80% cell confluency. For all experiments, cells below passage number 20 were used.

The BetaCoV/Australia/VIC01/2020 strain of SARS-CoV-2 was obtained from the Victorian Infectious Diseases Reference Laboratory, Melbourne (*43*), through Public Health England. This virus was passaged once in Vero cells for stocks used in this study. The virus was titrated in standard six-well plaque format on Vero cells, and one batch of virus was used for all experiments. Virus sequences were verified by deep sequencing.

### Transfection of Vero cells

The four structural proteins of SARS-CoV-2 were expressed in Vero cells using a pEVAC vector backbone. The day before transfection, Vero cells were seeded at 30% confluence in eight-well Ibidi μ-slides (catalog no. 80826) in antibiotic-free medium. Cells were transfected with Lipofectamine 3000 transfection reagent (Thermo Fisher Scientific) using 100 ng of plasmid DNA and 0.3 μl of Lipofectamine reagent per well. Cells were incubated for 48 hours under standard conditions before fixation and immunostaining as detailed below.

### Infection of Vero cells

The day before infection, Vero cells were seeded at 60% confluence in 24-well plates equipped with 13-mm round glass coverslips (VWR, catalog no. 631-0150). Cells were washed once with phosphate-buffered saline (PBS) before incubation with BetaCoV/Australia/VIC01/2020 diluted in PBS at a multiplicity of infection of 5. Incubation took place at room temperature (RT) on a rocking plate for 1 hour, whereupon inocula were removed and cells were washed twice with PBS and replenished with complete DMEM. Infection was allowed to progress under standard conditions (37°C, 5% CO_2_) for 0-, 5-, 7.5-, 10-, 12-, and 24-hour time periods. Cells were fixed with either formaldehyde (4% methanol-free formaldehyde in 100 mM cacodylate buffer) or glyoxal [4% glyoxal and 10% ethanol in acetate buffer (pH 5), as previously reported (*44*)] after the removal of spent media. Fixation was carried out at 37°C for 20 min.

### Plaque assay

Plaque assays were performed as previously described for SARS-CoV-1, with minor amendments (*45*, *46*). The day before infection, Vero cells were seeded at 30% confluence in six-well plates. These subconfluent monolayers were infected with 10-fold serial dilutions of each sample in duplicate, diluted in serum-free media, for 1 hour at RT on a rocking plate. After the removal of the inocula and washing with PBS, 3 ml of 0.2% agarose in virus growth media was overlaid, and the cells were incubated at 37°C for 72 hours. At this time, the overlay media were removed, and cells were washed with PBS and fixed overnight with 10% formalin. Fixed monolayers were stained with toluidine blue, and the plaques were counted manually.

### Polymerase chain reaction

The viral load of the media collected before cell fixation at 0-, 5-, 7.5-, 10-, 12-, and 24-hour time points after infection was measured and quantified via RT-qPCR. Total RNA extraction of the media was performed using the QIAamp Viral RNA Mini Kit (QIAGEN) following the manufacturer’s instructions. Five microliters of the RNA extraction final elution was reverse-transcribed to cDNA and amplified according to the manufacturer’s protocol using the TaqMan Fast Virus 1-Step Master Mix (Thermo Fisher Scientific). The primer pair was as follows: 5′CAGGTATATGCGCTAGTTATCAGAC-3′ (forward) and 5′-CCAAGTGACATAGTGTAGGCAATG-3′ (reverse). The probe used was as follows: 5′-[6FAM]AGACTAATTCTCCTCGGCGGGCACG[TAM]-3′ (Sigma-Aldrich). Analysis was performed using the Rotor-Gene 6000 Series Software 1.7 (Corbett Life Sciences, QIAGEN).

To generate RNA standards for qRT-PCR, a 97-nucleotide fragment of the spike ORF was cloned into the pJET1.2 vector (Invitrogen). Following linearization with Hind III, in vitro RNA transcripts were generated using the T7 RiboMAX Express Large Scale RNA Production System (Promega). Transcripts were purified (RNA Clean and Concentrator, Zymo Research) and the integrity was confirmed by gel electrophoresis.

### Immunostaining of fixed cells

Cells were fixed as detailed in the “Infection of Vero cells” section. The choice of fixative was determined by the structures to be immunostained in each sample, as detailed in table S1. A summary of the fixations and permeabilization conditions for the micrographs shown in this paper is reported in table S2. Fixed cells were incubated with 50 mM NH_4_Cl in PBS for 10 min to quench fixation. Cells were permeabilized with either 0.2% saponin or 0.2% Triton X-100 (see table S2) in PBS for 15 min and then blocked with 10% goat serum (Abcam) in PBS for 30 min (adding 0.2% saponin for saponin-permeabilized samples). Cells were incubated with primary and secondary antibodies for 1 hour at RT; antibodies were diluted in PBS containing 1% goat serum (adding 0.2% saponin for saponin-permeabilized samples) as detailed in the “Antibodies” section. Samples not meant for expansion microscopy were counterstained with DAPI (4′,6-diamidino-2-phenylindole) (Abcam, ab228549) diluted 1:1000 in PBS for 15 min at RT and mounted on Fisherbrand glass microscope slides (Fisher Scientific, catalog no. 11572203) using the VECTASHIELD Vibrance mounting reagent (2B Scientific).

### Expansion microscopy

The fixed immunostained samples were expanded following a published procedure (*47*) and imaged either on a confocal or on a light sheet microscope as previously reported (*48*). Briefly, immunostained cells were incubated with 0.25% glutaraldehyde in PBS for 15 min, washed three times with PBS, and then incubated with monomer solution (1× PBS, 2 M NaCl, 2.5% acrylamide, 0.15% *N*,*N*′-methylenebisacrylamide, and 8.625% sodium acrylate) for ~2 min at RT. Gelation was started inverting coverslips onto a drop of 150 μl of gelling solution [monomer solution/10% N,N,N′,N′-tetramethylethylenediamine (TEMED)/10% ammonium persulfate (APS), mixed in ratio 96:2:2] and left to gelate for 1 hour at RT in a humidified environment. Gels were digested in digestion buffer [1× tris-acetate-EDTA buffer (TAE), 0.5% Triton X-100, and 20 mM CaCl_2_] containing proteinase K (~8 U/ml) overnight at 37°C. Gels were eventually placed in double-distilled water to expand. The expansion factor (4.2) was calculated as previously reported (*48*).

### Microscopes

#### *Wide-field microscope*

Wide-field imaging of fixed SARS-CoV-2–infected cells was carried out on a custom-built automated wide-field microscope. Frame (IX83, Olympus), stage (Prior), Z drift compensator (IX3-ZDC2, Olympus), four-wavelength high-power light-emitting diode light source (LED4D067, Thorlabs), and camera (Zyla sCMOS, Andor) were controlled by Micro-Manager (*49*). Respective filter cubes for DAPI (filter set 49000-ET-DAPI, Chroma), Alexa Fluor 488 (filter set 49002-ET-EGFP, Chroma), Alexa Fluor 568 (filter set 49008-ET-mCherry, Texas Red, Chroma), Alexa Fluor 647 and Atto 647N (excitation filter 628/40, dichroic beam splitter Di02-R635, emission filter 708/75, Semrock), as well as Atto 490LS (filter set 49003-ET-EYFP, Chroma; emission filter replaced by 600LP, Semrock) were used. Images were acquired with an Olympus U Plan Apo 60×/1.42 NA (numerical aperture) oil objective lens at 30 to 35 random positions for each sample.

#### *Confocal microscopes*

The imaging of nonexpanded fixed samples was performed on a Zeiss LSM 800 microscope using a Plan-Apochromat 63×/1.4 NA oil objective. The microscope was controlled using the Zen software (version 2.6), and, for acquisition of 16-bit images, a pinhole size of 1.0 Airy unit (AU) for each channel, a scan speed of 5 (1.47 μs per pixel), and four times averaging were used. Pixel size was 70.6 nm. Expanded gels were cut to fit in a round glass-bottom dish (Ibidi μ-dish, catalog no. 81158) precoated with poly-l-lysine and were imaged on a Leica SP5 microscope using an apochromatic 63×/1.2 NA water objective. Images were acquired using a scanning frequency of 10 Hz and a pixel size ranging from 100 to 150 nm. To increase the collection of signal from the samples, the pinhole size was opened to 2.0 AU (in contrast to the preset value of 1.0 AU), which corresponds to an optical section of 1.5 μm.

#### *Light sheet microscope*

Expanded samples were imaged on a custom-built inverted selective plane illumination microscope (iSPIM). Parts were purchased from Applied Scientific Instrumentation (ASI) including the controller (TG8_BASIC), scanner unit (MM-SCAN_1.2), right-angle objective mounting (SPIM-K2), stage (MS-2K-SPIM) with motorized Z support (100-mm travel range; Dual-LS-100-FTP), and filter wheel (FW-1000-8). All components were controlled by Micro-Manager by means of the diSPIM plugin. The setup was equipped with a 0.3 NA excitation objective (10×, 3.5-mm working distance; Nikon) and a higher, 0.9 NA detection objective (W Plan-Apochromat 63×, 2.4 mm working distance; Zeiss) to increase spatial resolution and fluorescence signal collection. Lasers (OBIS445-75 LX, OBIS488-150 LS, OBIS561-150 LS, and OBIS647-120 LX, Coherent) were fiber-coupled into the scanner unit. An sCMOS camera (ORCA-Flash 4.0, Hamamatsu) was used to capture fluorescence. Respective emission filters were BrightLineFF01-474/27, BrightLineFF01-540/50, BrightLineFF01-609/54, and BrightLineFF0-708/75 (Semrock). Gels containing expanded samples were cut into small strips and mounted onto 24 mm by 50 mm rectangular coverslips with expanded cells facing upward using Loctite super glue (Henkel), as previously reported (*48*). The sample was then placed into an imaging chamber (ASI, I-3078-2450), which was filled with double-distilled water. We recorded volumes with a plane spacing of 0.5 μm. Raw data were deskewed using a custom MATLAB routine including a denoising step to remove hot pixels. Stacks were automatically separated in the respective color channels and were individually processed. Maximum intensity projections were generated of the deskewed stacks.

#### *Correlative structured illumination and atomic force microscope*

Correlative AFM/fluorescence microscopy measurements were performed as described before (*50*). AFM measurements were performed on a BioScope Resolve AFM (Bruker), operated in PeakForce Quantitative Nanoscale Mechanical Characterization (QNM) mode, which was combined with a custom-built structured illumination microscopy system (*51*). A 60×/1.2 NA water immersion lens (UPLSAPO 60XW, Olympus) was used for fluorescence excitation and detection, which was captured with an sCMOS camera (C11440, Hamamatsu). The wavelengths used for excitation were 488 nm (iBEAM-SMART-488, Toptica), 561 nm (OBIS 561, Coherent), and 640 nm [diode laser module (MLD), Cobolt]. Images were acquired using customized structure illumination microscopy (SIM) software.

### Deconvolution

Confocal images and deskewed light sheet microscopy data of expanded samples were deconvolved using the PSF Generator and DeconvolutionLab2 plugins in Fiji (*52*). In total, 25 to 100 iterations of the Richardson-Lucy algorithm were used. Deconvolved data were maximum intensity projected in Fiji, optionally using color to indicate depth.

### Replication kinetics from wide-field data

The percentage of cells expressing each of the structural proteins of SARS-CoV-2 and dsRNA was calculated semiautomatically using the image-processing program Fiji (*53*). Cells expressing SARS-CoV-2 proteins and dsRNA were counted manually, whereas the total number of cells was determined automatically using the “Analyze particles” function. Images of the DAPI-stained nuclei were filtered using the “Subtract background” (rolling ball radius = 20 pixels), “Gaussian blur” (sigma = 15 pixels), and “Unsharp Mask” (radius = 10 pixels, mask weight = 0.8) functions. Otsu thresholding was used to create a binary mask image. Dividing cells and cells at the edges of the image were excluded from analysis. For each time point, 30 to 35 wide-field microscope images (1000 to 1500 cells) were counted. For the control time point 0 hpi, only around 250 cells were counted. To determine the average expression levels of each of the structural proteins of SARS-CoV-2, the infected cells were segmented manually and the average fluorescence intensity in each viral protein channel was measured. From each value, the mean background intensity was subtracted and data were normalized using the highest average intensity value of the respective time point (usually at 12 or 24 hpi) for each protein. For each time point and protein, more than 80 cells were analyzed, except the early time point of 5 hpi with only around 40 cells because the fraction of infected cells was very low (5 hpi: *n* = 46, 7.5 hpi: *n* = 84, 10 hpi: *n* = 85, 12 hpi: *n* = 88, and 24 hpi: *n* = 81 for N, S, and M; and 5 hpi: *n* = 40, 7.5 hpi: *n* = 104, 10 hpi: *n* = 83, 12 hpi: *n* = 94, and 24 hpi: *n* = 82 for E).

### Stage kinetics from wide-field data

The OpenCV and scikit-image Python libraries were used for analysis. Quantification was performed on a dataset of ~35 wide-field images per time point stained for the SARS-CoV-2 N protein, the SARS-CoV-2 S protein, GM130 (Golgi apparatus), and nucleus (DAPI). Infection stages were assigned to each infected cell in the following way. First, binary masks for the cell nuclei were created by using local Otsu thresholding followed by contour detection and filtering (see “Fragmentation analysis” section). For N protein detection, global Otsu thresholding was applied to the corresponding channel. Then, for each detected nucleus, the nucleus masks were used to create thin perinuclear regions around the edge of each nucleus by upscaling each mask by a factor of 1.15 and subtracting the original mask, producing thin hoops around each nucleus. The cell was counted as containing the N protein if more than one pixel in this region was above the threshold value. The S protein analysis was the same, except a fixed threshold value was used instead of Otsu thresholding, and the masks were upscaled by a factor of 1.2 to produce a thicker perinuclear region, as S protein signal was sparser than that of the N protein. If cells were positive for N protein, but not S protein, they were classified as stage 1. To distinguish between stages 2 and 3, a fragmentation analysis of the Golgi apparatus (see “Fragmentation analysis” section) was performed.

The accuracy of the method was checked by manually counting the fraction of cells with the N protein and S protein signal as well as the fraction with a fragmented Golgi apparatus at ~10 to 15 images at 5 and 10 hpi. The results produced by the algorithm were within 1 SD of the mean values determined by manual analysis.

### Image segmentation

The OpenCV and scikit-image Python libraries were used for the segmentation. Wide-field and confocal images were segmented to enable cell-specific analysis of the dataset as follows. Initially, all channels of the images were merged to a grayscale image and the background was removed via Li thresholding (*54*). Connected component analysis was performed to segment the single-cell units in the image. To be segmented as an object of interest, a connected cluster was filtered via a minimum size of ~150 μm^2^ (corresponds to 30,000 pixels for confocal images; the size of a typical cell nucleus was ~200 to 250 μm^2^). In case of high cell density or staining of extended structures (e.g., microtubules), connected component analysis might lead to large numbers of cells being detected as one cluster. Here, when a maximum cell cluster size of 1,000,000 pixels was extended, the number of nuclei in the cluster was isolated using the DAPI-stained nuclei. For each nucleus in the cluster, the distances to its *K* nearest neighboring nuclei were measured (usually use *K* = 2 or *K* = 3, given that in most cases <10 cells make up one cluster). The cell outline of each single-cell unit was then defined by the outlines of the nucleus (DAPI channel) and the half-distances to its *K* nearest neighbors (choosing the maximum sized box that included all mentioned positions). For images characterized by low cell density, the described methods successfully segmented all cells that can be identified manually. For high–cell density images or including extended cell structures, these methods led to a good estimation of the cell outline for the majority of cells (>75% by visual inspection).

### Colocalization analysis

We quantified the spatial correlation between all four viral structural proteins by measuring Spearman’s rank coefficients. The Spearman coefficient is based on the ranking of image intensities. After assigning ranks to the pixel intensity values in each of the two channels, the Pearson correlation, which measures the degree of correlative variation, between the rank values of the pixel intensities in the two images is calculated. We also calculated the Manders coefficient that, in contrast to the Spearman coefficient, measures co-occurrence of intensities in the two channels rather than their correlation (*55*). However, interpretation of the Manders coefficient can be difficult because it depends on the ratio of total intensities in both channels. In contrast to the Spearman coefficient, the Manders coefficient is also affected by out-of-focus signal.

Spearman’s rank coefficients and Manders overlap coefficients were computed by ColocAnalyzer. ColocAnalyzer is a custom program for image filtering and colocalization analysis, which is free and available here: https://github.com/LAG-MNG-CambridgeUniversity/ColocAnalyzer. First, we saved images in such a manner that each of the channels of interest fell into one of three main colors: red, green, or blue. Then, we chose the two channels of interest (for example, red + blue or green + red) to be analyzed. For each image, Otsu thresholding was applied before computing colocalization coefficients on the remaining pixels with higher intensities.

Spearman’s rank coefficients were computed by ColocAnalyzer as

$$\rho =1-\frac{6\sum_{q}d_{q}^{2}}{n(n^{2}-1)}$$

Here, *d_q_* = rank [*I*1(*q*)] − rank [*I*2(*q*)] is the difference between ranks computed for pixel *q* in channel 1 and in channel 2 independently. *n* is the number of pixels that were analyzed. Because, after thresholding, a substantial fraction of pixels was blanked (would have zero intensity), we used only those pixels that had nonzero values in both channels to avoid an impact from black pixels.

The Manders overlap coefficient was computed by ColocAnalyzer using the formula provided in the original paper (*56*)

$$\text{MOC}=\frac{\sum_{q}^{\mathrm{Np}}I1(q)*I2(q)}{\sqrt{\sum_{q}^{\mathrm{Np}}I1{\left(q\right)}^{2}*\sum_{q}I2{\left(q\right)}^{2}}}$$

where *I*1(*q*) and *I*2(*q*) are the intensities of pixel *q* in the first and second channel, respectively. *Np* is the total number of pixels taken for analysis.

### Spot detection and analysis

N protein, dsRNA, and lysosome spot detection and analysis from microscopy images were performed by a customized MATLAB routine. For spot detection, we first applied median filtering to the image: Each pixel intensity value is decreased by a median value of intensities in a subarea of 60 pixels by 60 pixels around this pixel (the size of this subarea was chosen empirically). After Otsu thresholding of the filtered image, we determined the positions of the connected pixels with nonzero intensities. We called each cluster of such connected pixels a spot. Last, we filtered out spots that were smaller than 300 nm in diameter (approximately corresponds to Abbe’s resolution limit) and had a mean intensity value smaller than 10% of the maximum intensity. The area of each detected spot was calculated from the number of pixels per spot (the pixel size was 107.3 nm). For spot shape analysis, we fitted each spot to an ellipse with the customized MATLAB routine “fit_ellipse.m” (www.mathworks.com/matlabcentral/fileexchange/3215-fit_ellipse) and used the two radii *R*_min_ and *R*_max_ obtained from fitting to compute the eccentricity value of each spot: $e=\sqrt{1-{\left(\frac{R_{\text{min}}}{R_{\text{max}}}\right)}^{2}}$, where *R*_min_ and *R*_max_ are the smaller and larger radii of the ellipse, respectively. The distance between N protein and dsRNA spots was calculated as the minimal distance between the two spot centers.

The spot-to-spot distance between S protein and the lysosome marker LAMP1 was analyzed using the spot colocalization plugin ComDet for Fiji. For particle detection within the plugin, particle sizes between 3 and 4 pixels (corresponds to 210 to 280 nm) and an intensity threshold of 3 to 10 SDs of the average particle intensity were selected. The maximum distance between colocalized spots was set to 4 pixels (corresponding to 280 nm).

### Fragmentation analysis

Fragmentation analysis of the Golgi apparatus was performed on 16-bit wide-field images. The OpenCV and scikit-image Python libraries were used for the analysis. First, the channel with the DAPI-stained nuclei was segmented into cell nuclei and background using local Otsu thresholding followed by contour detection using the cv2.findContours function within the OpenCV library. The detected contours were filtered by size and circularity to ensure only the single nonoverlapping nuclei were selected. Specifically, contours with lengths in the range of 250 to 2500 pixels (30 to 300 μm) were accepted. From manual inspection, the nuclei contours fell roughly within the range of 300 to 700 pixels (35 to 85 μm). Only contours with length-to-area ratios of less than 0.05 were selected to eliminate non-elliptical shapes. The contours were then scaled down to 90% of their original size to avoid overlap with structures from other channels and filled to produce a mask for each image.

Next, local Otsu thresholding was performed on the Golgi apparatus channel. The mask of the corresponding nucleus was subtracted from the result. A rectangular region was created around each detected nucleus for subsequent location of the Golgi apparatus. The size of the region was determined by first creating a rectangle such that its borders were tangential to the outline of the detected nucleus and then scaling up its size by a factor of 2. Contour detection was performed within each region to locate the Golgi apparatus or its fragments. The angular size of each contour with respect to the center of the corresponding nucleus was calculated. It was found that a fragmented Golgi apparatus was typically detected as a single contour because thresholding of the wide-field images did not resolve the large number of small fragments, so only the size of the largest detected fragment for each corresponding nucleus was recorded. Contours with the angular size of less than 20° were found to be indistinguishable from noise, and so, the corresponding cells were excluded from the analysis.

### Microtubule directionality analysis

Directionality of microtubules was computed by a custom MATLAB routine on the basis of the texture detection technique introduced in (*57*). The method relies on computing gray-level co-occurrence matrices (GLCMs) as proposed in (*58*). The matrix is defined for single values of pixel position shifts [*dx*, *dy*] and consists of relative frequencies *p_ij_* that two pixels with gray levels *i* and *j* are separated by [*dx*, *dy*]. For eight-bit images, the GLCM will be a matrix of 256 × 256 elements. Instead of using [*dx*, *dy*], we used the concept of angle and distance: [φ, *d*]. We varied the distances from 10 to 100 pixels in 5-pixel steps (19 values). The minimum of 10 pixels corresponds to approximately 1 μm, so that short microtubules less than 1 μm in length were excluded from the analysis. The range of directions φ = [0°:180°] was divided into 45 segments with 4° steps for fine resolution of directionality. In total, we generated 19 × 45 = 855 GLCMs for each image. Then, as in (*57*), we computed the joint probability of occurrence for the specified pixel pair

$$T(\varphi ,d)=\sum_{i}\sum_{j}\frac{p_{\mathrm{ij}}(i-\mu_{x})(i-\mu_{y})}{\sigma_{x}\sigma_{y}}$$

where μ*_x_*, μ*_y_*, σ*_x_*, and σ*_y_* are the means and SDs of *p_x_* and *p_y_*, respectively: $p_{x}=\sum_{i}p_{\mathrm{ij}}$, $p_{y}=\sum_{j}p_{\mathrm{ij}}$. Next, we averaged those values across distances to leave only the angular dependence

$$T(\varphi )=\frac{\sum_{d}T(\varphi ,d)}{19}$$

Then, we obtained the texture correlation values *H*(φ) by normalizing the joint probability for each direction

$$H(\varphi )=\frac{T(\varphi )}{\sum_{i=0}^{45}T(4i)}$$

The texture correlation function shows greater values for the angles with preferable directions in microtubule images. Visual inspection on a number of microtubule images showed good performance of the method and its ability to find precisely (up to 4° in our case) dominating microtubule directions in the image. Last, the directionality coefficient was computed from summing up the second moments around each peak, from valley to valley

$$D=1-\gamma \sum_{p}^{n_{p}}\sum_{\varphi \epsilon w_{p}}[{\left(\varphi -\varphi_{p}\right)}^{2}H(\varphi )]$$

where *n_p_* is the number of peaks in *H*(φ), φ*_p_* is the value of an angle at the *p*th peak, *w_p_* is the range for the *p*th peak between two valleys, and γ is the normalizing coefficient $\gamma =\frac{1}{45}\frac{1}{\sum_{p}^{n_{p}}\sum_{\varphi \epsilon w_{p}}{\left(\varphi -\varphi_{p}\right)}^{2}}$.

### Cell stiffness measurement and analysis

For AFM cell stiffness measurements, Vero cells were plated at 60% confluence in 50-mm glass-bottom dishes (GWST-5040, WillCo Wells BV) the day before infection, infected, and fixed as described before. Live cell probes (PFQNM-LC, Bruker AFM probes) were used for all experiments. The probes were precalibrated for spring constant (nominal of 0.08 N/m), and deflection sensitivity was calibrated at the start of each experiment. The force applied to the cells was kept constant throughout the experiments, with typical values ranging between 150 and 300 pN. Force curves were fitted to a Hertz model

$$F=\frac{4\sqrt{R_{c}}}{3}\frac{E}{1-\nu^{2}}\delta^{3/2}$$

where *R*_c_ is the radius of tip curvature, *v* is the sample’s Poisson’s ratio, *E* is the Young’s modulus, and δ is the indentation depth. Curve fitting and Young’s modulus calculation were performed using nanoscope analysis.

### Data visualization and statistical analysis

Graphs were plotted with GraphPad Prism. Statistical significance between two values was determined using a two-tailed, unpaired Student’s *t* test (GraphPad Prism). In the figures, asterisks denote statistical significance as calculated by Student’s *t* test (*P < 0.05, **P < 0.01, ***P < 0.001, and ****P < 0.0001).

## Notes

View/request a protocol for this paper from *Bio-protocol*.
