# Supplementary material for: SARS-CoV-2 nucleocapsid protein adheres to replication organelles before viral assembly at the Golgi/ERGIC and lysosome-mediated egress
Source: Sci Adv. 2022 Jan 7;8(1):eabl4895. doi: 10.1126/sciadv.abl4895 (PMC10954198; doi:10.1126/sciadv.abl4895)
Supplement: Supplementary file 1 — Figs. S1 to S17 Tables S1 and S2 Legends for movies S1 to S4 [file sciadv.abl4895_sm.pdf]

Science Advances
AAAS

Supplementary Materials for

SARS-CoV-2 nucleocapsid protein adheres to replication organelles before viral assembly at the Golgi/ERGIC and lysosome-mediated egress

Katharina M. Scherer, Luca Mascheroni, George W. Carnell, Lucia C. S. Wunderlich, Stanislaw Makarchuk, Marius Brockhoff, Ioanna Mela, Ana Fernandez-Villegas, Max Barysevich, Hazel Stewart, Maria Suau Sans, Charlotte L. George, Jacob R. Lamb, Gabriele S. Kaminski-Schierle, Jonathan L. Heeney, Clemens F. Kaminski*

*Corresponding author. Email: cfk23@cam.ac.uk

Published 7 January 2022, *Sci. Adv.* **8**, eabl4895 (2022)
DOI: 10.1126/sciadv.abl4895

The PDF file includes:

Figs. S1 to S17
Tables S1 and S2
Legends for movies S1 to S4

Other Supplementary Material for this manuscript includes the following:

Movies S1 to S4

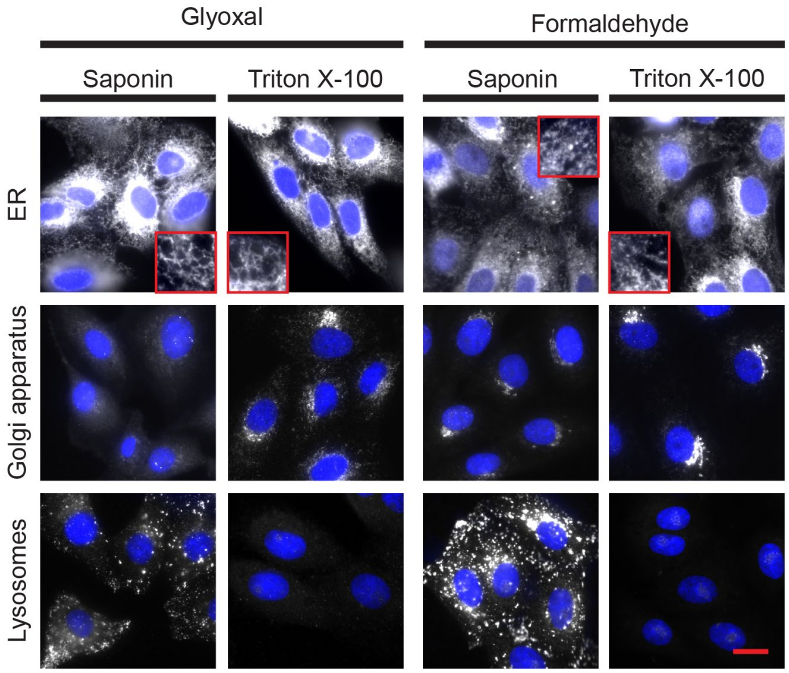

Fig. S1. Optimal fixation and immunostaining conditions differ for host cell organelles. The immunostaining of three different cell structures (endoplasmic reticulum (ER), Golgi apparatus and lysosomes) in fixed Vero cells using different chemical fixatives (formaldehyde, glyoxal) and different detergents (triton X-100, saponin) shows that each structure has its own optimal fixation and permeabilisation conditions. The ER was stained using an anti-calnexin antibody; the Golgi apparatus was stained using an anti-GM130 antibody; the lysosomes were stained using an anti-LAMP1 antibody. All primary antibodies were detected using the same Alexa Fluor 488-conjugated secondary antibody. White channel: cellular structures; blue channel: cell nuclei. In the red square: detail of the tubular region of the ER. All pictures shown were acquired on a custom-built widefield microscope. Scale bar: 20 µm. The size of the micrograph inserts is 12 µm per side.

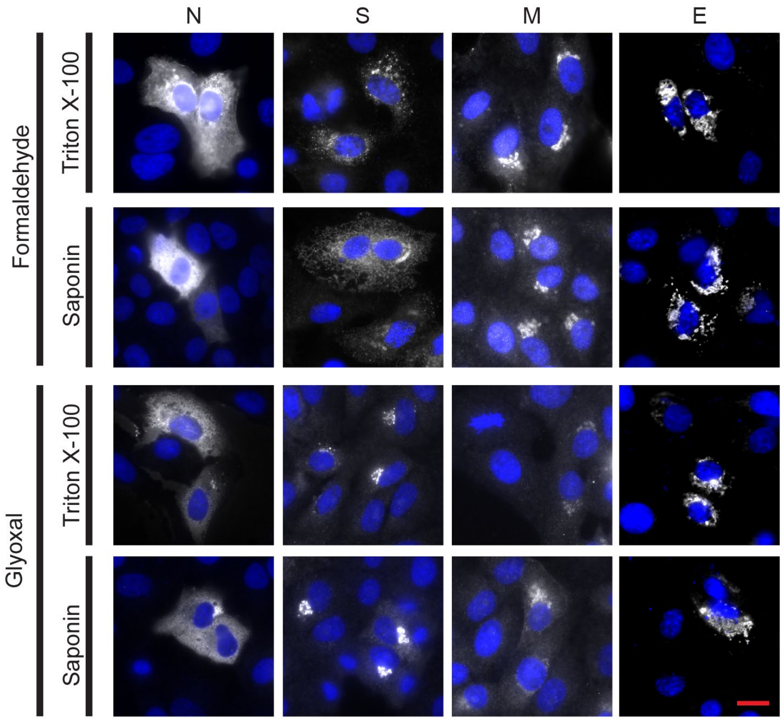

Fig. S2. Immunostaining of the four structural proteins of SARS-CoV-2 (nucleocapsid (N), spike (S), membrane (M) and envelope (E)) was successful for all fixation conditions and selected antibodies.

Vero cells were transfected to express each of the four viral proteins. The immunostaining was successful for all chemical fixation (formaldehyde or glyoxal) and permeabilisation (triton X-100 or saponin) conditions tested. The spike protein pattern was different in the two fixation conditions tested, although this difference was not noticed in the infected samples. Nucleocapsid protein was detected using an Alexa Fluor 568-conjugated secondary antibody; spike, membrane and envelope proteins were detected using Alexa Fluor 488-conjugated secondary antibodies. White channel: SARS-CoV-2 proteins; blue channel: DAPI-stained nuclei. All pictures shown were acquired on a custom-built widefield microscope. Scale bar: 20 µm.

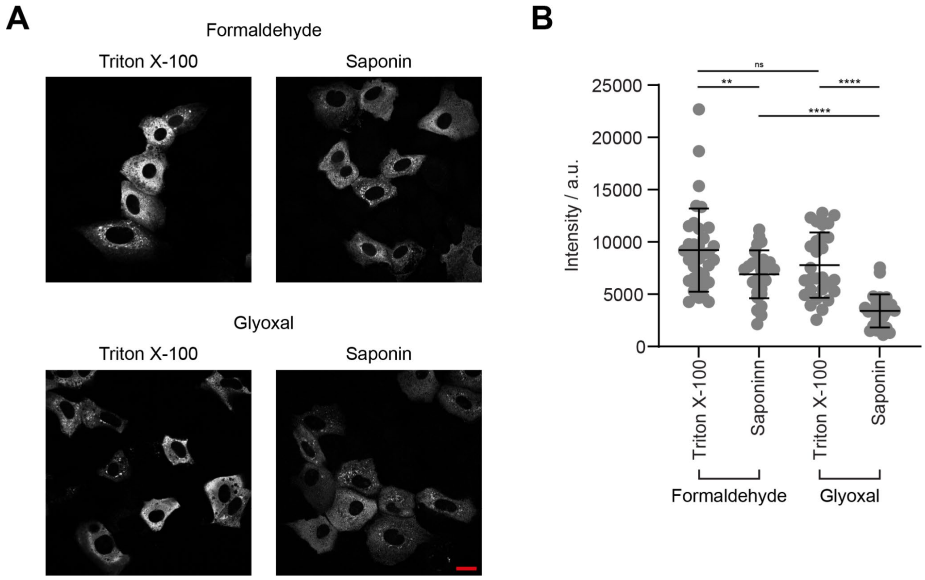

Fig. S3. Fixation and permeabilisation conditions influence signal intensity of dispersed, cytoplasmic N protein.

A) Representative confocal images for each condition. Scale bar: 20 µm. B) Cytoplasmic intensity of N protein was measured in at least 25 cells per condition.

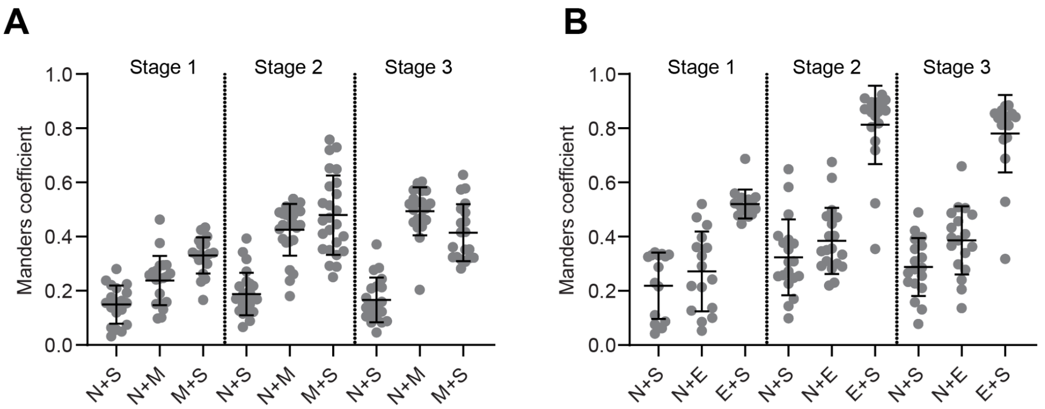

Fig. S4. The SARS-CoV-2 spike (S), membrane (M) and envelope (E) proteins highly colocalise, while the nucleocapsid (N) protein shows lower colocalisation with the other viral proteins.

A) Colocalisation analysis between N, S and M at different infection stages determined using the Manders coefficient method. B) Colocalisation analysis between N, S and E at different infection stages determined using the Manders coefficient method.

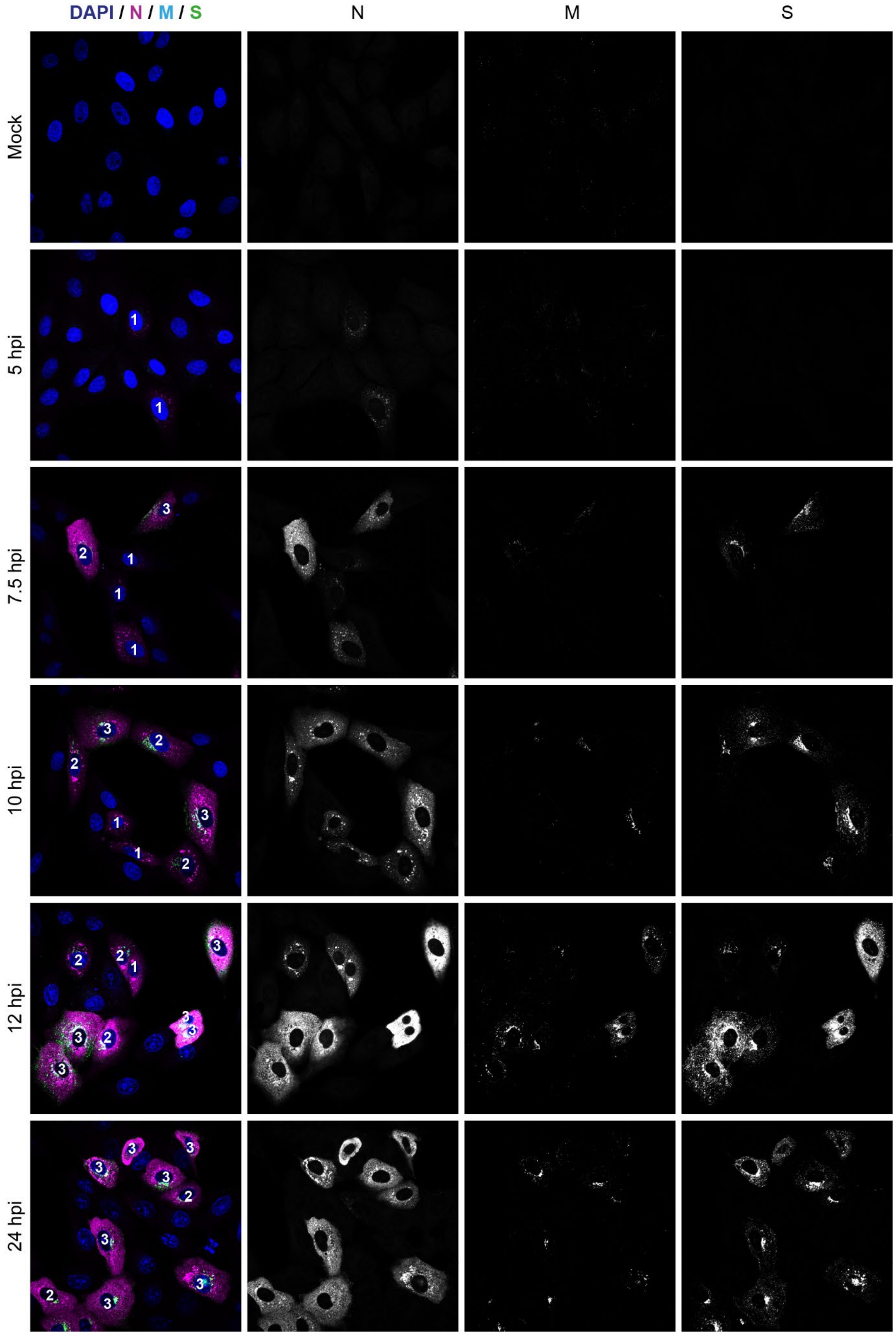

Fig. S5. Exemplary images for every time point of the experiment and the mock infected control.

Cells were fixed with glyoxal and stained for N, M and S proteins through permeabilisation with saponin. Magenta: nucleocapsid protein (N); cyan: membrane protein (M); green: spike protein (S); blue: nuclei. In addition to composite images, single channel images are shown. Numbers indicate if cells are stage 1, 2 or 3 of infection. The size of the micrographs is 200 µm per side.

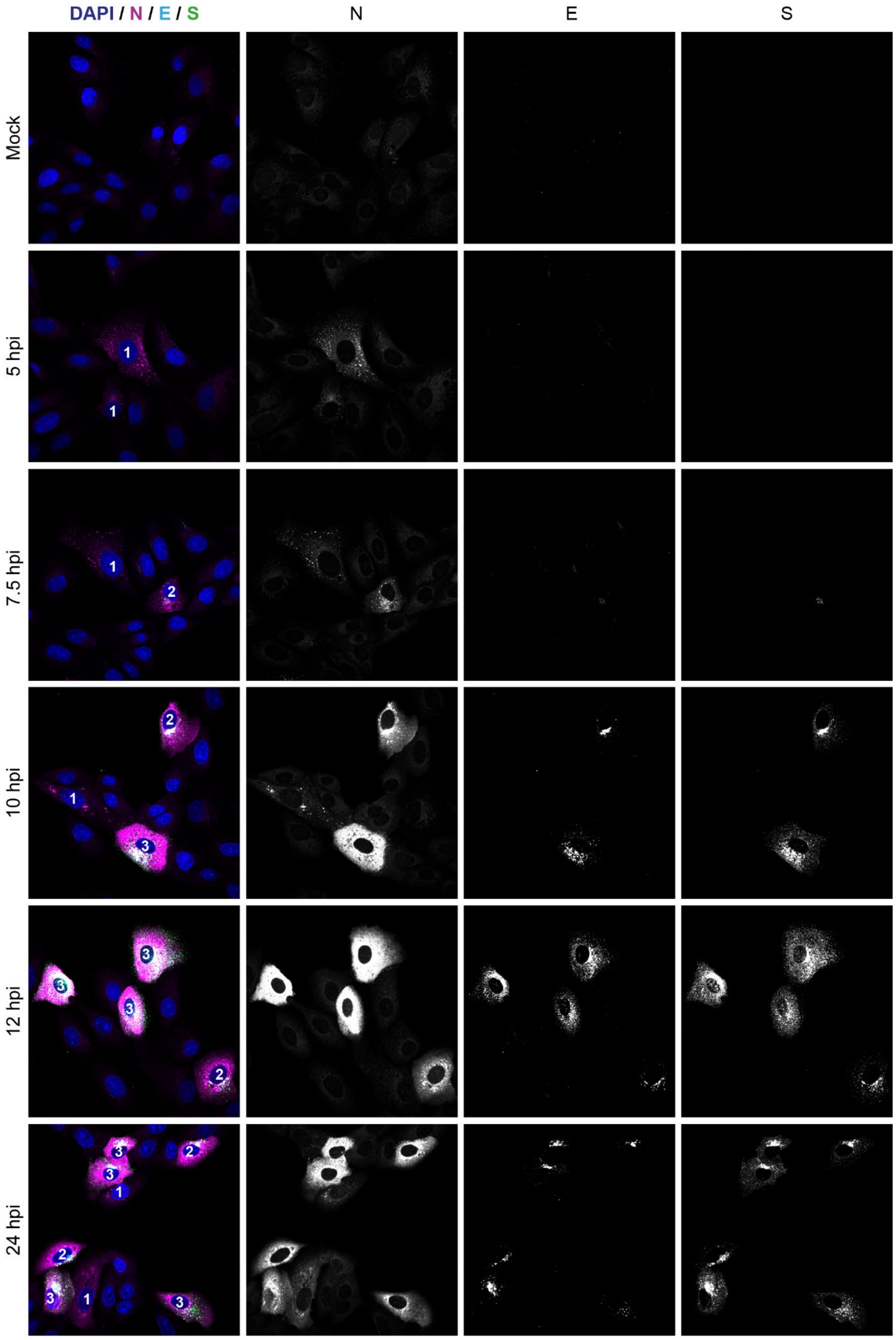

Fig. S6. Exemplary images for every time point of the experiment and the mock infected control.

Cells were fixed with glyoxal and stained for N, E and S proteins through permeabilisation with Triton X-100. Magenta: nucleocapsid protein (N); cyan: envelope protein (E); green: spike protein (S); blue: nuclei. In addition to composite images, single channel images are shown. Numbers indicate if cells are stage 1, 2 or 3 of infection. The size of the micrographs is 200 µm per side.

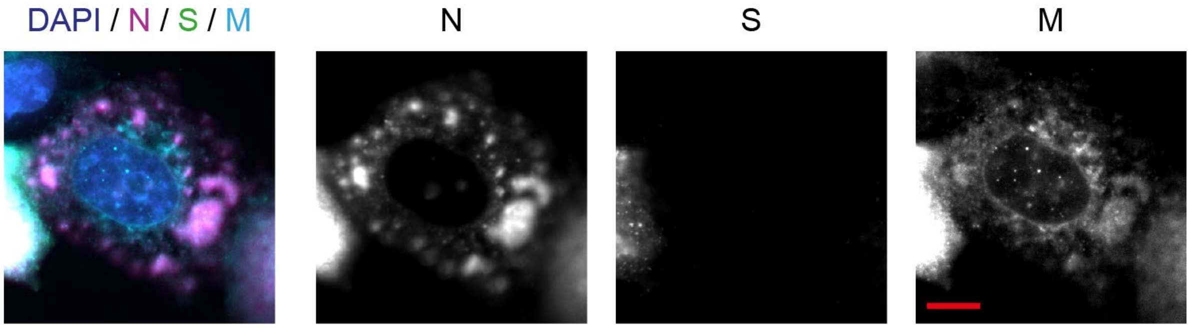

Fig. S7. The SARS-Cov-2 membrane protein is expressed slightly earlier than the spike protein in infected Vero cells.

In ~5% of cases, infected cells were expressing the membrane protein but not the spike protein, indicating slightly different expression kinetics. Images were acquired on a custom-built widefield microscope. Magenta: nucleocapsid protein (N); green: spike protein (S); cyan: membrane protein (M); blue: nuclei. Scale bar: 10 µm.

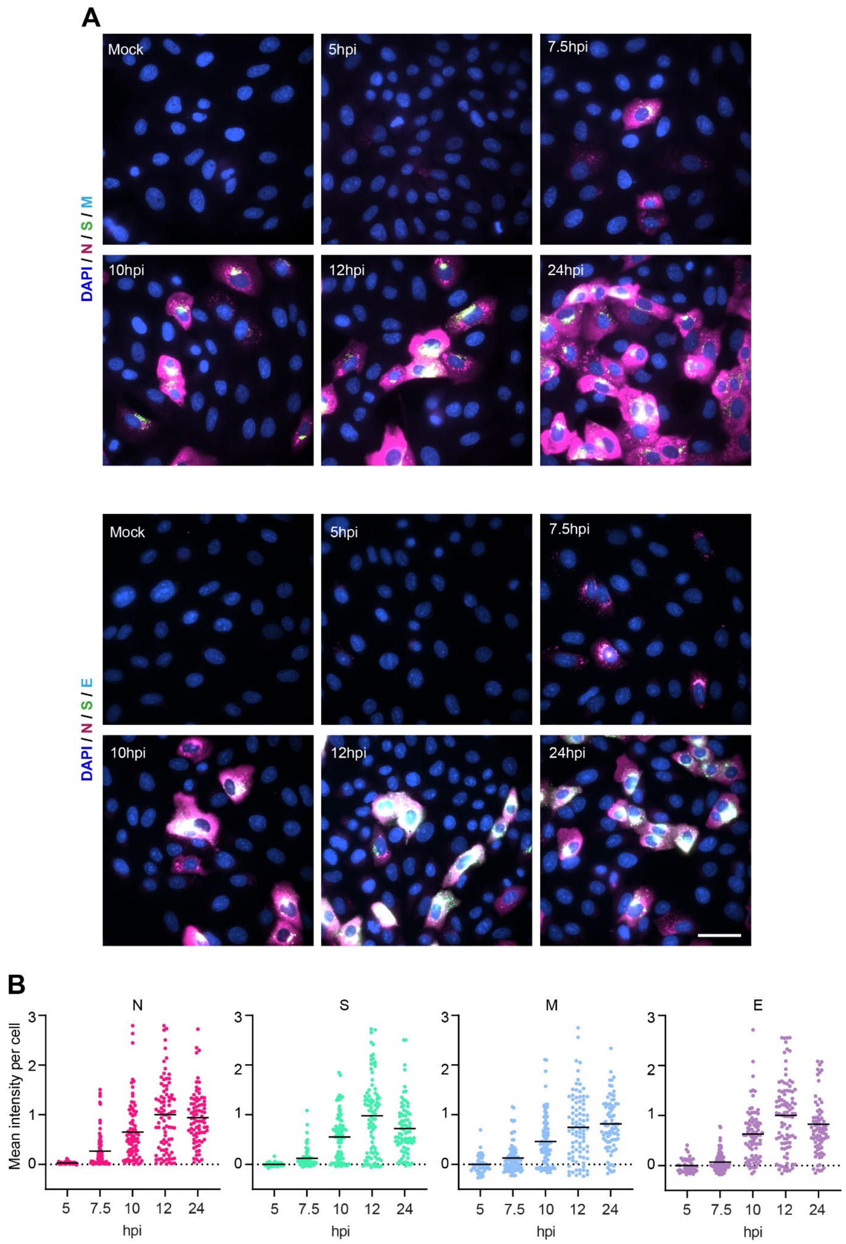

Fig. S8. Expression of the four structural SARS-CoV-2 proteins (N, S, M and E) in infected Vero cells saturates at 10 hpi.

A) Representative widefield microscopy images of SARS-CoV-2 infected Vero cells, fixed and immunostained at a series of time points post infection. Magenta: nucleocapsid protein (N); green: spike protein (S); cyan: membrane protein (M); blue: nuclei. Scale bar 50 µm. B) The images were used to determine the expression levels of each of the SARS-CoV-2 structural proteins by measuring the average fluorescence intensity per infected cell. Each data point in the graphs represents one cell. N is expressed from 5 hpi onwards, while the other proteins (S, M , E) are expressed concurrently from 7 hpi. The expression of all four structural proteins increases almost linearly up to 12 hpi, when it plateaus, suggesting an equilibrium between newly synthesized viral proteins and released virions.

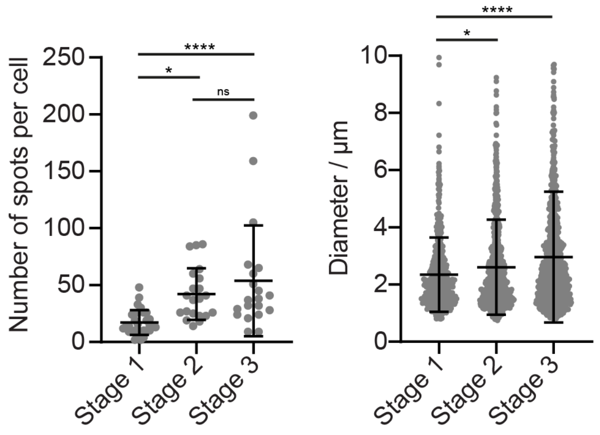

Fig. S9. The average number of N compartments per cell as well as their size increase as the infection progresses.

Left graph: stage 1: n = 30, stage 2: n = 21, stage 3: n = 30. Right graph: stage 1: n = 515, stage 2: n = 887, stage 3: n = 1076.

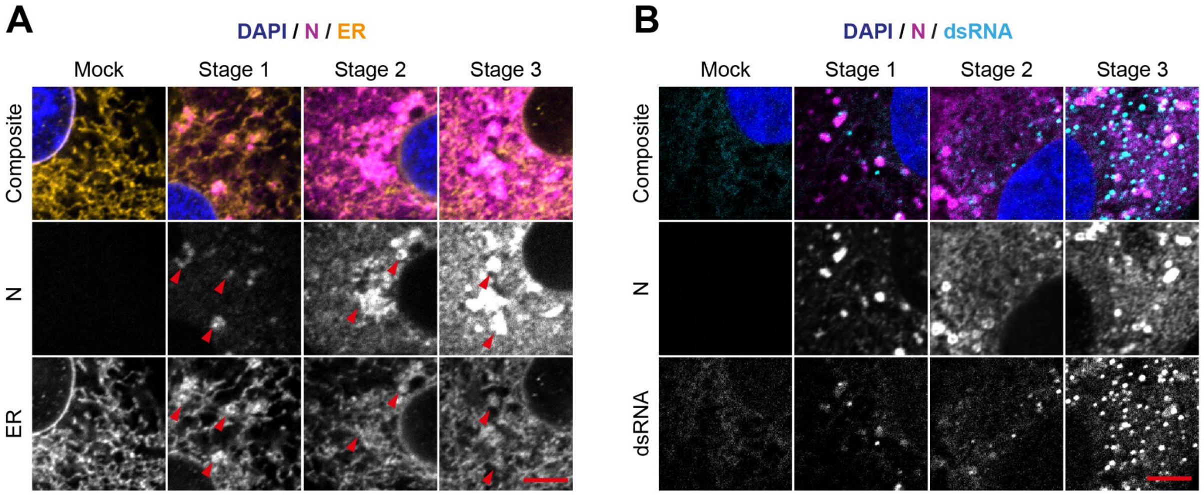

Fig. S10. Nucleocapsid protein (N) foci are connected to the endoplasmic reticulum, but only partially overlap with RNA replication foci.

A) Confocal images of cells in all three infection stages show that the N compartments (magenta) form at regions of condensed ER (orange) and are tethered to it at all stages of infection. Scale bar 5 µm. B) Confocal images of cells in all three infection stages show that the RNA replication foci (cyan) increase in number as the infection progresses and that they partially overlap with the compartments formed by the nucleocapsid protein (magenta). Scale bar 5 µm.

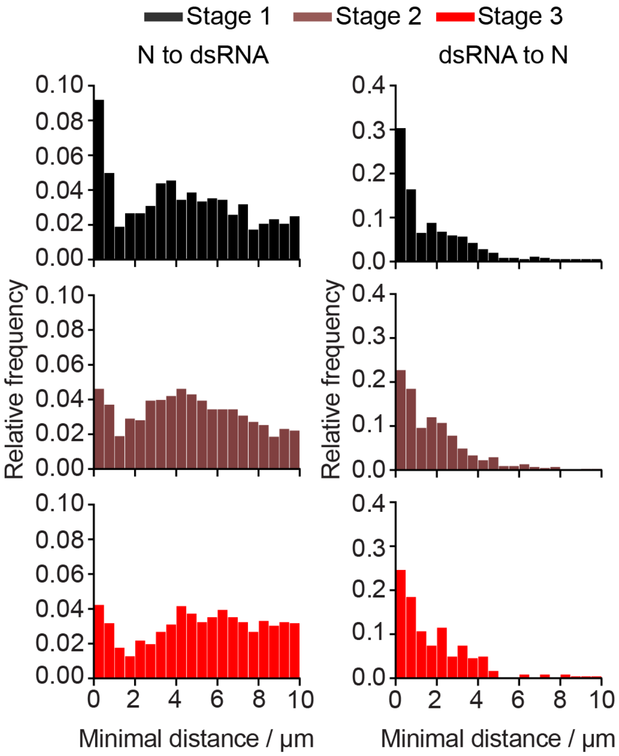

Fig. S11. Minimum distance analysis between N compartments and dsRNA foci.
The fraction of closely associated (within <1 µm) N compartments and dsRNA foci decreases during stages 2 and 3.

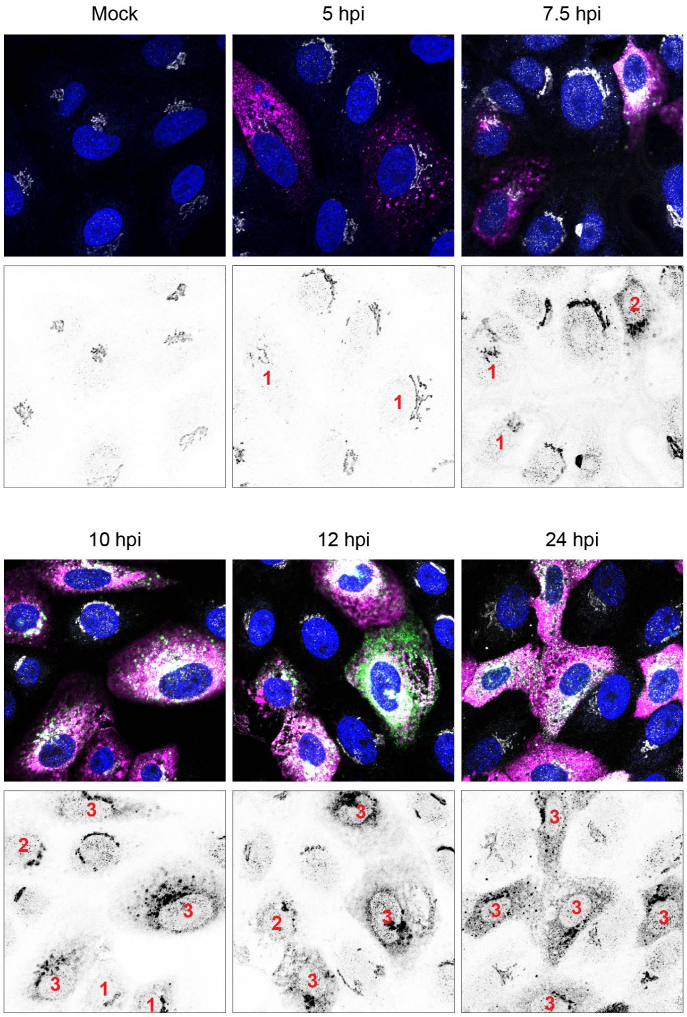

Fig. S12. Exemplary images for every time point of the experiment and the mock infected control.

Cells were fixed with formaldehyde and stained for N and S proteins as well as the Golgi apparatus (GM130) and nuclei through permeabilisation with saponin. Magenta: nucleocapsid protein (N); green: spike protein (S); cyan: Golgi (GM130); blue: nuclei. In addition to composite images, single channel images of the Golgi are shown in an inverted greyscale to accentuate the structures. Numbers indicate if cells are stage 1, 2 or 3 of infection. The size of the micrographs is 100 µm per side.

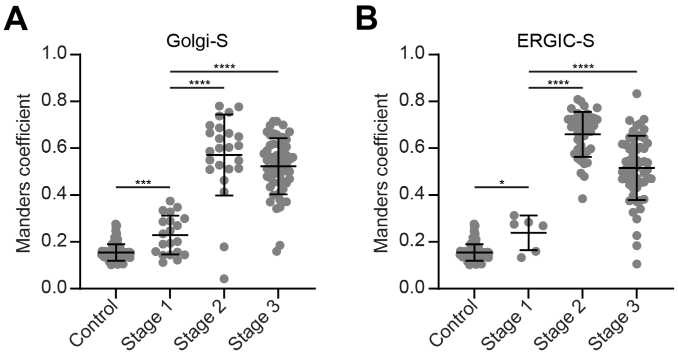

Fig. S13. The spike protein (S) of SARS-CoV-2 colocalises with both the Golgi apparatus and the ER to Golgi compartment (ERGIC) in infected Vero cells.

A) Colocalisation analysis (determined using the Manders coefficient method) between the Golgi apparatus and S shows partial spatial correlation from the moment S starts to be expressed (stage 2) onwards. B) Colocalisation analysis (determined using the Manders coefficient method) between the ERGIC and S shows partial spatial correlation from the moment S starts to be expressed (stage 2) onwards. At least 20 cells were analysed for each infection stage and organelle except for ERGIC-stage 1, where only six cells were analysed. Significance was tested with an unpaired t-test with Welch's correction for unequal standard deviations.

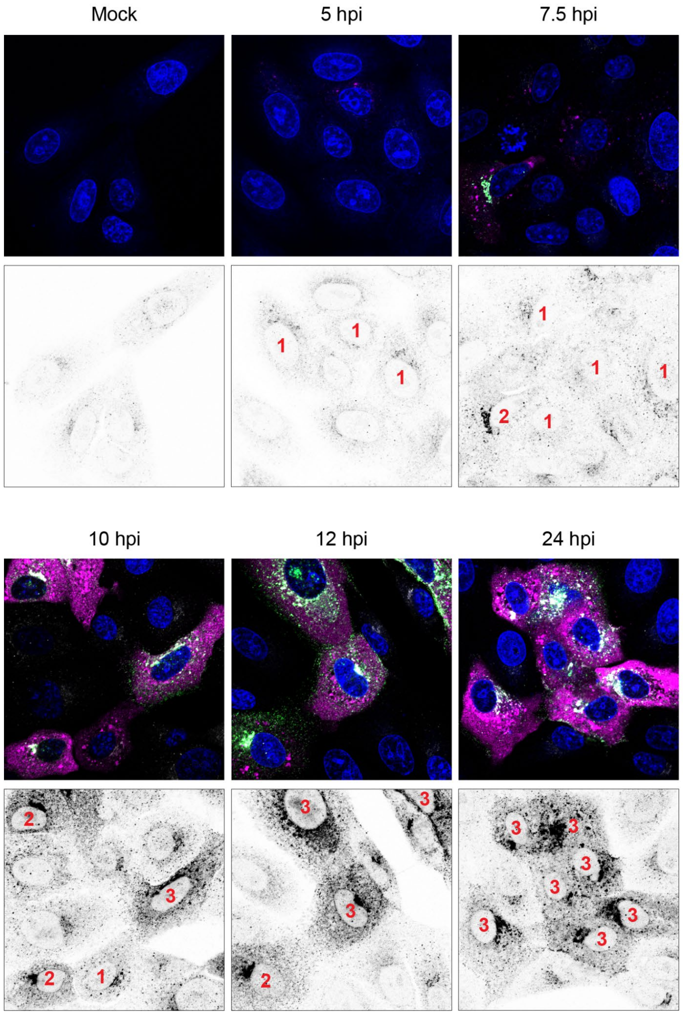

Fig. S14. Exemplary images for every time point of the experiment and the mock infected control.

Cells were fixed with glyoxal and stained for N and S proteins as well as the ERGIC (LMAN1) and nuclei through permeabilisation with saponin. Magenta: nucleocapsid protein (N); green: spike protein (S); cyan: ERGIC (LMAN1); blue: nuclei. In addition to composite images, single channel images of the ERGIC are shown in an inverted greyscale to accentuate the structures. Numbers indicate if cells are stage 1, 2 or 3 of infection. The size of the micrographs is 100 µm per side.

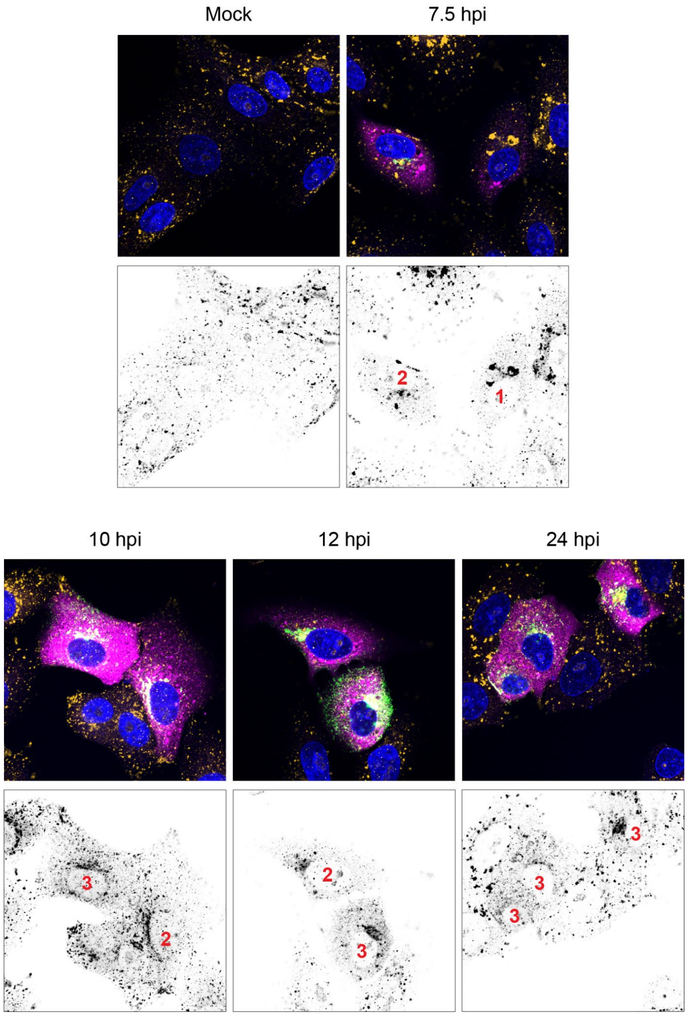

Fig. S15. Exemplary images for every time point of the experiment and the mock infected control.

Cells were fixed with glyoxal and stained for N and S proteins as well as the lysosomes (LAMP1) and nuclei through permeabilisation with saponin. Magenta: nucleocapsid protein (N); green: spike protein (S); yellow: lysosomes (LAMP1); blue: nuclei. In addition to composite images, single channel images of the lysosomes are shown in an inverted greyscale to accentuate the structures. Numbers indicate if cells are stage 1, 2 or 3 of infection. The size of the micrographs is 100 μm per side.

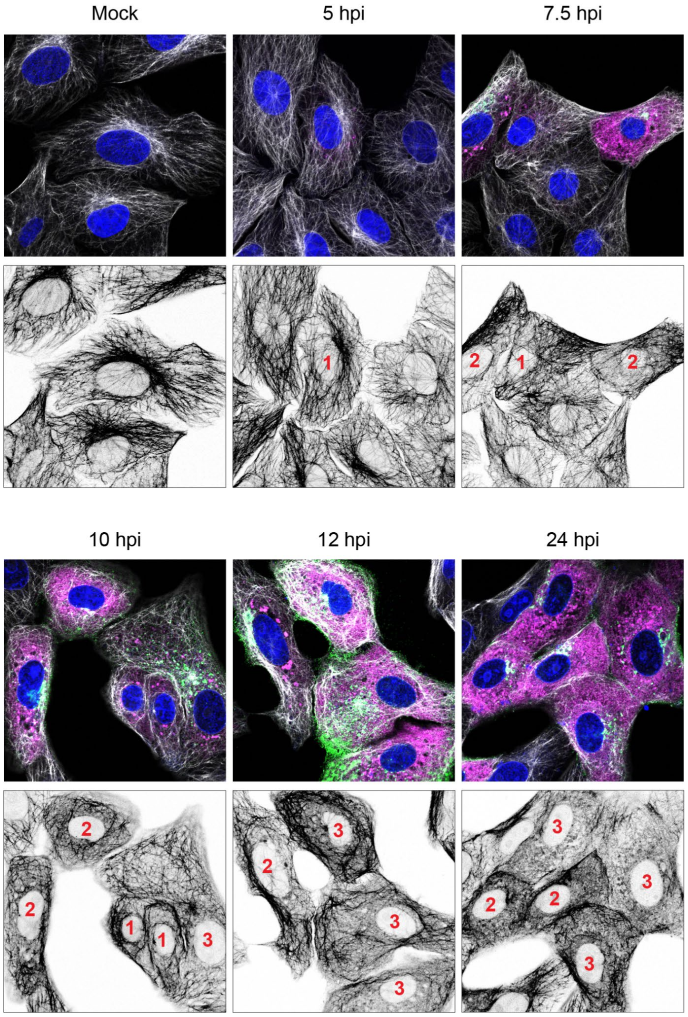

Fig. S16. Exemplary images for every time point of the experiment and the mock infected control.

Cells were fixed with glyoxal and stained for N and S proteins as well as the microtubules (β-tubulin) and nuclei through permeabilisation with saponin. Magenta: nucleocapsid protein (N); green: spike protein (S); grey: microtubules (β-tubulin); blue: nuclei. In addition to composite images, single channel images of the microtubules are shown in an inverted greyscale to accentuate the structures. Numbers indicate if cells are stage 1, 2 or 3 of infection. The size of the micrographs is 100 µm per side.

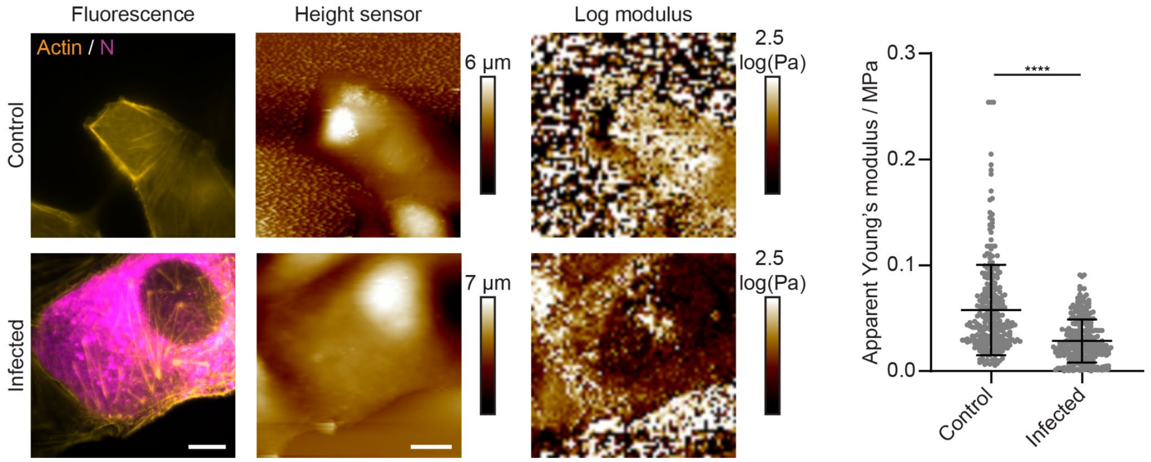

Fig. S17. The apparent Young's modulus is reduced in infected cells which indicates a softening of the probed cell surface compared to non-infected cells.

Correlative fluorescence and atomic force microscopy was performed on SARS-CoV-2 infected cells fixed at 12 hpi. Staining of the actin cytoskeleton and of the SARS-CoV-2 nucleocapsid protein (N) was used to distinguish between infected and non-infected cells. The Young's modulus was obtained by fitting the individual force curves in each picture. Each data point in the graph represents one force curve; for each condition, force curves were obtained from 8 independent cells. Scale bars: 10 µm. Significance was tested using a Mann-Whitney test.

	Formaldehyde		Glyoxal	
	Saponin	Triton X-100	Saponin	Triton X-100
SARS nucleocapsid protein	✓	✓	✓	✓
SARS Spike protein	✓	✓	✓	✓
SARS Membrane protein	✓	✓	✓	✓
SARS Envelope protein	✓	✓	✓	✓
Double-stranded RNA	✗	✓	✗	✓
ER (calnexin)	✓	✗	✓	✗
Golgi (GM130)	✓	✓	✗	✗
ERGIC (LMAN1)	✗	✗	✓	✗
Lysosomes (LAMP1)	✓	✗	✓	✗
Microtubules (beta-tubulin)	✓	✓	✓	✓
Microfilaments (actin)	✗	✓	✗	✗

Table S1.

Optimised fixation and permeabilisation conditions for the immunostaining of the structures imaged in this work. A tick represents a working combination, while a cross represents a non-working combination.

Figure	Fixation	Permeabilisation
Fig. 1A, stages 1 and 3, Fig. 1B	Glyoxal	Saponin
Fig. 1A, stage 2, Fig. 1C	Formaldehyde	Triton
Fig. 2A, 2B, 2C, 2E, 2F, 2G	Glyoxal	Saponin
Fig. 3A, 3C	Glyoxal	Saponin
Fig. 3B	Formaldehyde	Triton
Fig. 4A	Formaldehyde	Saponin
Fig. 4B, 4C	Glyoxal	Saponin
Fig. 5B, 5C	Glyoxal	Saponin
Supp. Fig. 1, 2, 3	As indicated	As indicated
Supp. Fig. 4A)	Glyoxal	Saponin
Suppl. Fig. 4B	Formaldehyde	Triton
Suppl. Fig. 5B, 5C	Glyoxal	Saponin
Suppl. Fig. 6	Formaldehyde	Triton
Suppl. Fig. 7	Glyoxal	Saponin
Suppl. Fig. 8 (top)	Glyoxal	Saponin
Suppl. Fig. 8 (bottom)	Formaldehyde	Triton
Suppl. Fig. 10A	Glyoxal	Saponin
Suppl. Fig. 10B	Formaldehyde	Triton
Suppl. Fig. 12	Formaldehyde	Saponin
Suppl. Fig. 14	Glyoxal	Saponin
Suppl. Fig. 15	Glyoxal	Saponin
Suppl. Fig. 16	Glyoxal	Saponin
Suppl. Fig. 17	Formaldehyde	Triton

Table S2.

Summary of fixation and permeabilisation conditions for all the samples shown in the figures of the manuscript.

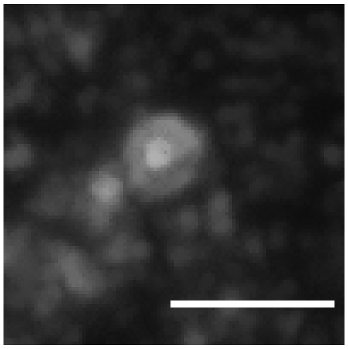

Movie S1.

The video shows a single N protein compartment consisting of two layers. SARS-CoV-2 infected Vero cells were fixed, immunostained, expanded and imaged on a light sheet microscope. Scale bar 1.2 µm (taking into account an expansion factor of 4.2).

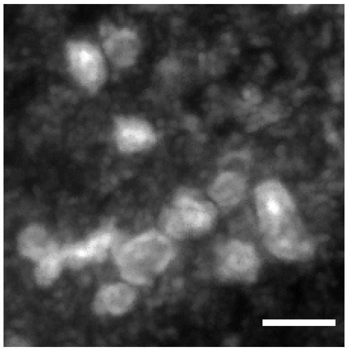

Movie S2.

Multiple N protein compartments, partially fused and consisting of two layers. SARS-CoV-2 infected Vero cells were fixed, immunostained, expanded and imaged on a light sheet microscope. Scale bar 1.2 µm (taking into account an expansion factor of 4.2).

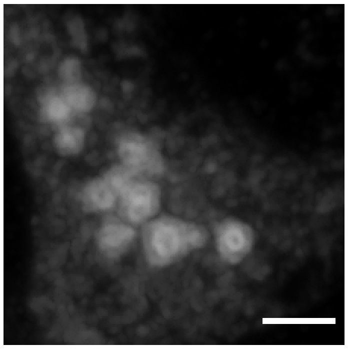

Movie S3.

Multiple N protein compartments, partially fused and consisting of two layers. SARS-CoV-2 infected Vero cells were fixed, immunostained, expanded and imaged on a light sheet microscope. Scale bar 1.2 µm (taking into account an expansion factor of 4.2).

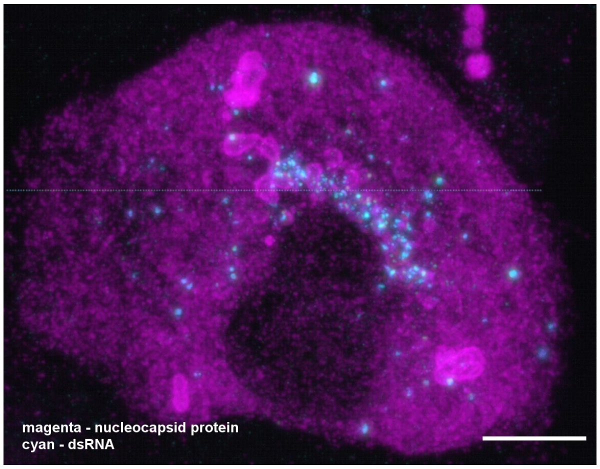

Movie S4.

A combination of expansion and light sheet microscopy reveals the spatial arrangement between dsRNA (cyan) and the nucleocapsid protein N (magenta) in SARS-CoV-2 infected Vero cells. The video corresponds to Figure 3G in the main manuscript. The majority of dsRNA foci are located in a region immediately adjacent to the nucleus. In addition, most N compartments contain dsRNA foci sitting in the layers of the compartments. Scale bar 5 µm (taking into account an expansion factor of 4.2).
